# The near-complete genome assembly of allotetraploid *Pennisetum purpureum* ‘Purple’ reveals the genetic and epigenetic landscape of centromeres

**DOI:** 10.1093/hr/uhaf301

**Published:** 2025-10-29

**Authors:** Yongji Huang, Jinbin Lin, Jun Xu, Xinyi Lin, Zuhu Deng, Xiaoxian Zhong, Sheng Zuo, Zhiliang Zhang

**Affiliations:** Fujian Key Laboratory on Conservation and Sustainable Utilization of Marine Biodiversity, College of Geography and Oceanography, Minjiang University, Fuzhou, China; Fujian Key Laboratory on Conservation and Sustainable Utilization of Marine Biodiversity, College of Geography and Oceanography, Minjiang University, Fuzhou, China; Center for Plant Biology, School of Life Sciences, Tsinghua University, Beijing, China; State Key Laboratory of Crop Gene Resources and Breeding, Institute of Crop Sciences, Chinese Academy of Agricultural Sciences (CAAS), Beijing, China; National Nanfan Research Institute, Chinese Academy of Agricultural Sciences (CAAS), Sanya, Hainan, China; Fujian Key Laboratory on Conservation and Sustainable Utilization of Marine Biodiversity, College of Geography and Oceanography, Minjiang University, Fuzhou, China; National Engineering Research Center for Sugarcane, Fujian Agriculture and Forestry University, Fuzhou, Fujian, China; National Engineering Research Center for Sugarcane, Fujian Agriculture and Forestry University, Fuzhou, Fujian, China; Institute of Animal Science, Jiangsu Academy of Agricultural Sciences, Nanjing, China; Anhui Provincial Key Laboratory of Molecular Enzymology and Mechanism of Major Metabolic Diseases, College of Life Sciences, Anhui Normal University, Wuhu, China; State Key Laboratory of Crop Gene Resources and Breeding, Institute of Crop Sciences, Chinese Academy of Agricultural Sciences (CAAS), Beijing, China; National Nanfan Research Institute, Chinese Academy of Agricultural Sciences (CAAS), Sanya, Hainan, China

## Abstract

Drastic karyotype changes are a major evolutionary force, potentially involving centromere position, number, distribution, or strength alterations. Yet, the genetic and epigenetic landscape of centromeres, especially in allopolyploid plants during subgenome reshuffling, remains poorly understood. Here, we present a near-complete chromosome-scale genome assembly of the allotetraploid *Pennisetum purpureum* ‘Purple’, resolving all 14 centromeres. We find that subgenome-biased expansion of six LTR retrotransposons drives architectural divergence between subgenomes. Centromeric satellite repeats (CentPs) show rapid sequence divergence across subgenomes and chromosomes, with CENH3 preferentially binding conserved higher order repeats. Intriguingly, centromeric retrotransposons in *Pennisetum* (CRPs) are evolutionarily younger compared to their noncentromeric counterparts, coupled with marked subgenome B-biased amplification. Notably, CRP insertions flanking CentP satellites correlate with elevated satellite DNA polymorphism, supporting a model wherein CentP homogenization processes actively purge retrotransposons from centromeric arrays. Despite rapid sequence diversification of centromeric repeats, the epigenetic landscapes remain evolutionarily conserved in the centromeres of two subgenomes. Additionally, comparative analyses across *Pennisetum* species demonstrate rapid species- and chromosome-level turnover of CentPs and CRPs. Overall, our study illuminates the genetic and epigenetic plasticity of centromeres in allopolyploids, revealing how centromeric repeats adapt post-subgenome reshuffling.

## Introduction

Centromeres are indispensable chromosomal elements in eukaryotes, which fulfill a crucial role in ensuring precise chromosome segregation during cellular division [1]. Although these regions are generally composed of satellite repeats and retrotransposons, their functional identity is epigenetically determined by the incorporation of CENH3, a centromere-specific histone variant that replaces canonical histone H3 [2]. Despite their conserved function, centromere sequences evolve rapidly, exhibiting significant diversity across different species [1]. For instance, centromeres in rice (*Oryza sativa*) are characterized by a 155-bp satellite repeat sequence CentO, yet this sequence is either lost or replaced by different centromeric satellite repeats in several closely related *Oryza* species [3, 4]. A growing body of experimental evidence suggests a complex role for the centromere in shaping the genome, such as through centromere-mediated translocations ‘shuffling’ the karyotype and other gross chromosomal rearrangements [5]. Nevertheless, the centromere sequence variation in the context of chromosomal rearrangements remains poorly understood, largely due to the challenges in assembling complete centromere sequences.

Allopolyploidization, an evolutionarily significant process pervasive across eukaryotic lineages, represents a key driver of plant speciation and crop domestication [6–9]. Allopolyploidization may induce genomic instability by triggering subgenomic variations [10, 11]. Notably, nascent polyploids demonstrate accelerated centromere evolution following the merger of divergent parental genomes [12–14]. Despite its significance, the adaptation of centromeres to the ‘genome shock’ triggered by allopolyploidization remains poorly understood, representing a critical gap in plant genome evolution.

Elephant grass (*Pennisetum purpureum*, syn. *Cenchrus purpureus*, 2*n* = 4*x* = 28, A’A’BB) is a perennial grass belonging to the genus *Pennisetum* in the tribe Paniceae of the family Poaceae [15]. This species is characterized by high biomass production, rapid growth, and broad adaptability, making it a valuable forage crop in tropical and subtropical regions [16]. Its cultivation represents an efficient strategy for utilizing marginal lands unsuitable for conventional crops and addresses increasing demands for animal-derived food products [17]. Genomic evidence suggests that elephant grass originated approximately 6.61 million years ago (MYA) through hybridization between its diploid A’ and B genome progenitors, followed by extensive genomic rearrangements [18]. Comparative genomic analyses reveal that the A’ subgenome shares closer homology with the A genome of pearl millet (*P. glaucum*, 2*n* = 2*x* = 14) [18]. However, due to the presence of numerous repetitive sequences, the assembly of the centromeric regions of elephant grass has been challenging. In recent years, advancements in sequencing technologies such as PacBio HiFi and ultra-long Oxford Nanopore Technology (ONT) have enabled the completion of telomere-to-telomere (T2T) genome assemblies for species like humans, rice, Arabidopsis, and maize [19–22]. Nevertheless, the two elephant grass genome assemblies released in 2021 and 2022, constrained by the read length and accuracy of ONT technology at the time, still contain numerous gaps and errors, particularly in centromeric and telomeric regions [18, 23]. Generating a complete genome sequence is crucial for both basic and applied research on elephant grass, as well as for elucidating its complex centromeric landscape.

Here, we present Purple-CEN, a significantly improved *de novo* assembly of the allotetraploid *P. purpureum* ‘Purple’ genome, generated by integrating high-fidelity PacBio HiFi reads with Hi-C chromatin interaction data. Compared to the two previously published elephant grass genomes, the Purple-CEN assembly exhibits superior completeness, contiguity, and accuracy, enabling the first complete resolution of all 14 centromeres in this species. Our comprehensive analysis reveals the genetic and epigenetic architecture of centromeres in the genus *Pennisetum*, providing novel insights into the diversity and evolution of centromeric regions in allopolyploid grasses.

## Results

### A near-complete genome assembly of allotetraploid *P. purpureum* ‘Purple’

**Figure 1:**
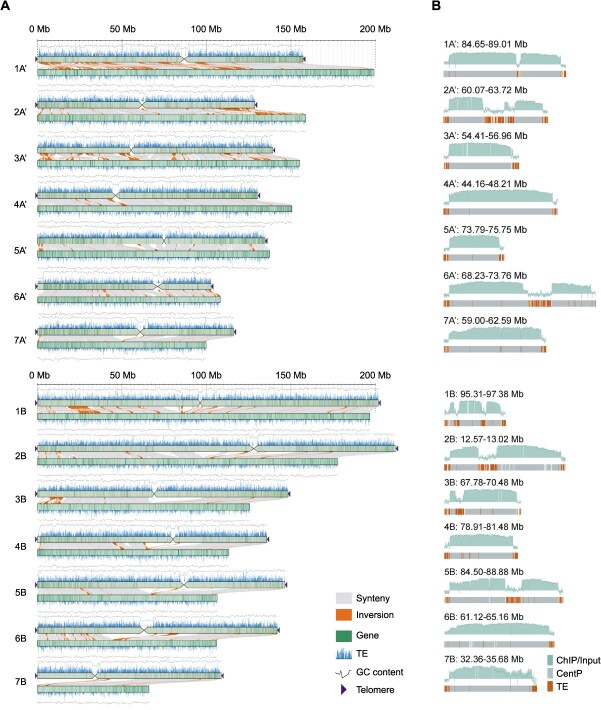
Synteny and the delimiting of centromeres in *P. purpureum* ‘Purple’. (A) Collinearity analysis between Purple-CEN and Purple-LZU. The collinearity between the two genome assemblies is shown as gray lines or blocks, and the inversions are shown in orange. The purple triangles indicate the presence of telomere sequence repeats. TE distribution is plotted above or under each chromosome in 100-kb bins, and the gene density in 100-kb windows is shown as green bars. (B) The delimiting of Purple-CEN centromeres. The layers of each chromosome graph indicate (1) the density of read mapping from CENH3 ChIP-seq with sliding windows of 10 kb shown in light blue, respectively; (2) the CentP satellite distribution; and (3) TE distribution, respectively. The dotted frame represents the defined centromere region.

To achieve a high-quality genome assembly for *P. purpureum* ‘Purple’, we first conducted a *k*-mer-based genome survey, which estimated the genome size to be approximately 2077.62 Mb with a heterozygosity level of 1.20%. (Fig. S1). We then generated 94.83 Gb of PacBio HiFi reads (approximately 45.65× coverage; Table S1) and performed initial assembly using HiCanu (v2.2) and NextDenovo [24, 25], yielding a preliminary assembly with a contig N50 of 133.5 Mb (Table 1). Using Hi-C scaffolding, we anchored 96.48% of contigs onto 14 pseudochromosomes, leaving only 10 unresolved gaps, with nine T2T pseudochromosomes fully assembled (Table 1, Fig. S2). Chromosome identification numbers and orientations were further refined and assigned to two subgenomes (SubA’ and SubB) based on the previously published allotetraploid *P. purpureum* ‘Purple’ genome and subgenome-specific *k*-mers using SubPhaser (Fig. S3) [26]. Subsequently, we utilized the published elephant grass genome to fill seven gaps within the preliminary assembly and to correct two telomeres. Using the seven-base telomeric motif (‘CCCTAAA/TTTAGGG’) as a query sequence, we successfully identified 25 out of the 28 telomeric regions across all 14 pseudochromosomes (Figs 1A and S4; Table S2). Ultimately, we obtained a near-complete reference genome of *P. purpureum* ‘Purple’ encompassing three gaps, with a total size of 2004.54 Mb and a chromosome N50 of 142.03 Mb (Table 1; Tables S2 and S3).

**Table 1:** Summary of genome assembly and annotation of *Pennisetum purpureum* ‘Purple’

**Category**	**Purple-CEN**	**Purple-LZU**
Assembly feature		
Total length of contigs	2077.62 Mb	1966.92 Mb
Total number of contigs	1166	2059
Anchor ratio of contigs	96.48%	96.65%
Assembled genome size	2004.54 Mb	1901.03 Mb
Number of telomeres	25	0
Number of gaps	3	1836
Contig N50	133.50 Mb	1.82 Mb
Chromosome N50	142.03 Mb	150.58 Mb
Mapping ratio of HiFi reads	99.97%	-
QV	60.04	22.59
BUSCO	99.20%	97.80%
LAI	13.51	3.25
Annotation		
Length of repetitive sequences	1284.97 Mb	1092.41 Mb
Percentage of repetitive sequences	64.10%	57.46%
Length of retrotransposons	659.73 Mb	560.69 Mb
Percentage of retrotransposons	32.91%	29.49%
Length of DNA transposons	70.50 Mb	69.34 Mb
Percentage of DNA transposons	3.52%	3.65%
Number of protein-coding genes	66 961	65 927

We employed multiple strategies to comprehensively assess the quality of the *P. purpureum* ‘Purple’ genome assembly. Initially, to validate the completeness of the assembly, we remapped the original PacBio HiFi sequencing data to the newly assembled genome, achieving a mapping rate of 99.97% (Table 1). Furthermore, the quality value (QV) of the assembled genome was 60.04 (Table 1), indicating a high base accuracy. The Long Terminal Repeat (LTR) Assembly Index (LAI) was 13.51 (Fig. S5; Table 1), suggesting high-assembly integrity even in regions rich in repetitive sequences. Benchmarking Universal Single-Copy Orthologs (BUSCO) assessment revealed a high gene completeness of 99.20% (Table 1). Hence, we concluded that the near-complete genome assembly of *P. purpureum* ‘Purple’ exhibits high continuity, completeness, and accuracy. Approximately, 64.10% of the genome was characterized as repetitive sequences, including 32.91% retrotransposons and 3.52% DNA transposons (Table 1). Through an integrated approach combining *de novo* prediction, homology-based searches, and transcriptome-guided methods, we annotated a total of 66 961 protein-coding genes (Table 1). These genes were predominantly enriched in the chromosomal arms, while transposable elements (TEs) were scattered throughout all chromosomes (Fig. 1A; Table 1).

To determine the precise locations and sequences of functional centromeres within the near-complete genome assembly of *P. purpureum* ‘Purple’, we performed chromatin immunoprecipitation (ChIP) using an anti-CENH3 antibody, which specifically recognizes the CENH3 in this accession (Fig. S6A). Based on CENH3 occupancy profiles, we accurately delineated the boundaries of core centromeres on each chromosome within the *P. purpureum* ‘Purple’ genome, which varied in size from 1.96 Mb (CEN5A’) to 5.53 Mb (CEN6A’) (Figs 1B and S7B). Most centromeres exhibited uniform HiFi read coverage in the CENH3-enriched regions without detectable breakpoints (Fig. S7), providing robust evidence for the continuity and high quality of all 14 centromeres. Additionally, the distribution of CENH3 density was found to be variable and uneven, with some centromeres binding CENH3 predominantly on centromeric satellite repeats (e.g., Cen3A’, Cen4A’, CEN5A’, Cen7A’, CEN4B, CEN6B, and CEN7B), whereas others showed a primary loading of CENH3 on both centromeric satellite repeats and TEs (e.g., CEN1A’, Cen2A’, Cen6A’, Cen1B, CEN2B, CEN3B, and CEN5B) (Fig. 1B). Given the high-quality centromere assembly, we designated this version as Purple-CEN, while referring to the previously published Lanzhou University version as Purple-LZU.

The near-complete genome of *P. purpureum* ‘Purple’ provided an opportunity to assess the enhancement in its genomic quality. Comparative analysis between the Purple-CEN and Purple-LZU versions revealed good collinearity across all chromosomes (Figs 1A and S8). However, we observed that large regions of homology were disrupted on chromosomes 1A’, 2A’, 3A’, and 1B in the Purple-LZU version, with the assembly of the short arm of chromosome 7B being omitted (Figs 1A and S8). The newly assembled Purple-CEN genome (2004.54 Mb) showed a 103.51 Mb increase compared to the Purple-LZU version (1901.03 Mb) (Table 1). This increase was primarily attributed to the superior assembly of repetitive sequences enabled by PacBio long-read technology, which added 192.56 Mb of newly assembled sequences, as confirmed by QV and LAI values for all chromosomes (Fig. S5; Tables 1 and S4). Notably, the Purple-CEN assembly annotated 66 961 protein-coding genes, representing an increase of 1034 genes compared to Purple-LZU (65 927 genes) (Table 1). In Purple-CEN, both the contig N50 and the longest contig had shown significant improvements, increasing from 1.82 to 133.50 Mb and from 15.07 to 212.15 Mb, respectively, representing a 73.35-fold and 14.08-fold increase (Table 1). Particularly noteworthy was Purple-CEN’s successful resolution of 25 telomeric sequences, with only three gaps remaining outside centromeric regions (Tables S2 and S3). In contrast, the Purple-LZU version lacked all telomeric sequences and contained 1836 unresolved gaps (Fig. S4; Table 1).

### Subgenome-specific abundant LTR retrotransposon proliferation shaped the distinct subgenome architectures of *P. purpureum* ‘Purple’

**Figure 2:**
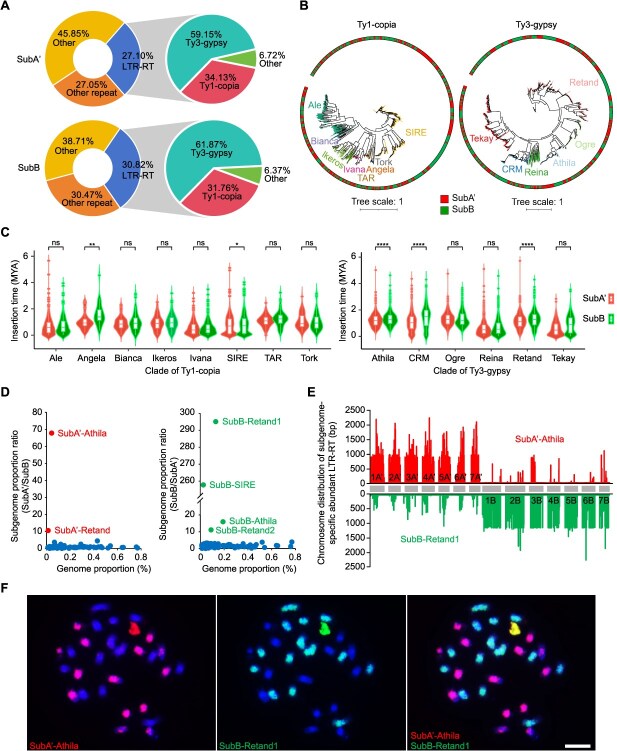
Transposable element (TE) dynamics between two subgenomes of *P. purpureum* ‘Purple’. (A) Repeat sequence distribution patterns and LTR-RT types. (B) Classification of LTR-RT superfamilies. The subgenome location of Ty1-copia and Ty3-gypsy and the subgenomes are indicated by the outer circle. (C) Estimated times of intact Ty1-copia and Ty3-gypsy insertion in two subgenomes of *P. purpureum* ‘Purple’. (D) Characterization of SubA’-specific and SubB-specific LTR-RTs in *P. purpureum* ‘Purple’. The y-axis is the subgenome proportion ratios. It represents the relative enrichment of the corresponding LTR-RT in the corresponding subgenome. The x-axis is the genome proportion for each LTR-RT in the genome of *P. purpureum* ‘Purple’. (E) Chromosome distribution of subgenome-specific abundant LTR-RTs. (F) FISH mapping of two subgenome-specific abundant LTR-RTs in *P. purpureum* ‘Purple’. Chromosomes counterstained with DAPI. FISH signals of probes SubA’-Athila and SubB-Retand2 in red and green color, respectively. Scale bar = 5 μm.

TEs are ubiquitous genomic components and one of the major forces driving genome evolution [27]. Given the extensive chromosomal reshuffling between subgenomes in *P. purpureum* ‘Purple’, we sought to understand how LTR retrotransposons (LTR-RTs) diverged across subgenomes. In the genome of *P. purpureum* ‘Purple’, we identified 237.13 Mb (accounting for 27.10% of SubA’) and 317.45 Mb (comprising 30.82% of SubB) of LTR-RTs in SubA’ and SubB, respectively (Fig. 2A). Among these, Ty3-gypsy elements were more prevalent than Ty1-copia, representing 59.15% and 61.87% of LTR-RTs in SubA’ and SubB, respectively (Fig. 2A).

To investigate the contributions of distinct LTR-RT lineages to two subgenomes, we performed a comprehensive analysis of full-length LTR-RTs. We performed phylogenetic analyses and identified eight clades within the Ty1-copia lineage (Angela, Ale, Bianca, Ikeros, Ivana, TAR, Tork, and SIRE) and six major branches in the Ty3-gypsy lineage (Tekay, CRM, Reina, Athila, Ogre, and Retand), all distributed across both subgenomes (Fig. 2B). While all lineages were shared between subgenomes, specific subfamilies expanded disproportionately in one subgenome (Fig. 2B). By estimating LTR-RT insertion times, we further discovered that SubB accumulated these elements earlier than SubA (Fig. 2C). Notably, particular subfamilies within these lineages exhibited a significantly earlier marked proliferation within SubB. Specifically, within the Ty1-copia lineage, subfamilies Angela and SIRE, and within the Ty3-gypsy lineage, Athila, CRM, and Retand expanded substantially in SubB between 0.12 and 2.00 MYA (Fig. 2C).

To analyze the genomic distribution of dominant LTR-RT subfamilies in *P. purpureum* ‘Purple’, we quantified their abundance variation between subgenomes. We identified subgenome-specific abundant LTR-RTs with a subgenome proportion ratio exceeding 10, and selected two potential SubA’-enriched and four SubB-enriched LTR-RTs (Fig. 2D). BLAST analysis against the EDTA-annotated TE library of *P. purpureum* ‘Purple’ (retaining hits with >90% identity and coverage) revealed that the two SubA’-enriched LTR-RTs exhibited high similarity to Athila and Retand of the Ty3-gypsy family, while the four SubB-enriched LTR-RTs included two Retand (Ty3-gypsy), one Athila (Ty3-gypsy), and one SIRE (Ty1-copia) (Fig. 2D; Table S5). Consequently, we designated these LTR-RTs as SubA’-Athila, SubA’-Retand, SubB-Retand1, SubB-Retand2, SubB-Athila, and SubB-SIRE, respectively (Fig. 2D).

To examine their chromosomal distribution, we mapped these LTR-RTs to the Purple-CEN genome. Chromosomal mapping demonstrated higher abundance of these LTR-RTs in their respective subgenomes, albeit with distinct distribution patterns (Figs 2E and S9). Athila and SIRE were dispersed across the chromosomes, with major peaks in centromeric and pericentromeric regions, whereas Retand was predominantly distributed across all chromosomes of the corresponding subgenome (Figs 2E and S9). We employed six subgenome-specific abundant LTR-RT probes for FISH on metaphase chromosomes of *P. purpureum* ‘Purple’. Distinct and intense hybridization signals were detected on the corresponding seven chromosome pairs for all probes except SubB-SIRE (Figs 2F and S10), indicating the capability of these LTR-RTs to differentiate chromosomes of the *P. purpureum* subgenomes.

In summary, our findings demonstrate that the distinct subgenome architectures of *P. purpureum* ‘Purple’ are shaped by the proliferation of subgenome-specific LTR-RTs.

### Rapid divergence of centromeric satellite repeats in the subgenomes of *P. purpureum* ‘Purple’

**Figure 3:**
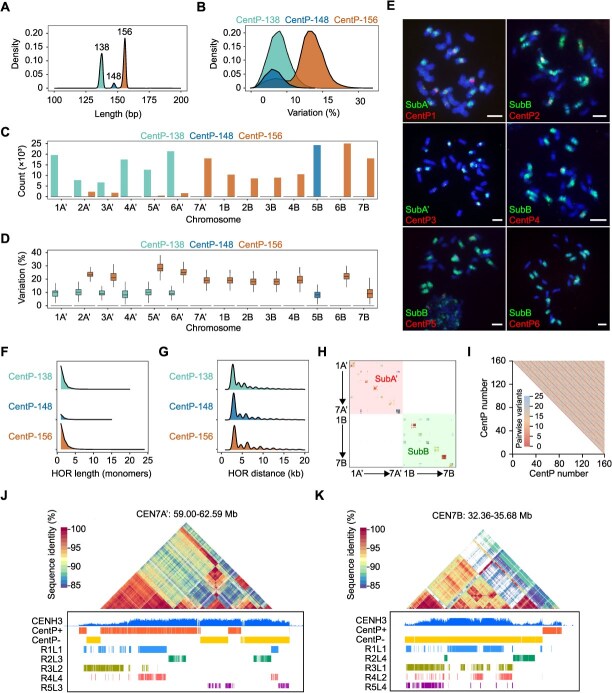
Evolution of centromeric satellite repeats in the subgenomes of *P. purpureum* ‘Purple’. (A) CentP monomer lengths (bp) in the genome of *P. purpureum* ‘Purple’. (B) Sequence variation of CentP monomer in the genome of *P. purpureum* ‘Purple’. (C) CentP count in all the chromosomes of the two subgenomes. (D) Sequence variation of CentP monomer in all the chromosomes of the two subgenomes. (E) FISH mapping of six CentP monomers in *P. purpureum* ‘Purple’. Chromosomes counterstained with DAPI. FISH signals of six CentP probes in red color, and corresponding subgenome-specific abundant LTR-RTs in green color. Scale bar = 5 μm. (F) The lengths (bp) of HOR blocks (monomers) in the genome of *P. purpureum* ‘Purple’. (G) The distance between CentP HORs (kb) in the genome of *P. purpureum* ‘Purple’. (H) Chromosome-specific distribution of CentP HORs. (I) Heatmap of a representative region within CEN1A’, shaded according to pairwise variants between CentP. (J and K) The distribution of CentPs and their HORs in CEN7A’ and CEN7B.

Typically, centromeric regions encompass megabase-scale repetitive sequences that facilitate the occupancy and loading of CENH3 [1, 20–22, 28]. Through the analysis of ChIP-seq data, we observed that the enrichment level of CENH3 within the centromeric satellite repeats in *Pennisetum* (CentP) was significantly higher than that in the chromosomal arm regions (Fig. 1B), with an average ChIP/input ratio reaching 27.12-fold (Table S6), indicating the specific enrichment of CENH3 in the centromeric regions. The 14 centromeres of *P. purpureum* ‘Purple’ are predominantly enriched with three different lengths of CentP monomers (138, 148, and 156 bp) (Fig. 3A), designated as CentP-138, CentP-148, and CentP-156. CentP-156 exhibited higher polymorphism than CentP-138 and CentP-148 (Fig. 3B), suggesting its more ancient evolutionary origin.

We conducted a more refined examination of the CentP monomer number and distribution across subgenomes. SubA’ contained more CentP satellite repeats (109 651 copies) than SubB (105 637 copies) (Fig. 3C; Table S7). Both subgenomes shared CentP-156 monomers localized at multiple centromeres: CEN2A’, CEN3A’, CEN5A’, CEN6A’, and CEN7A’ in SubA’, and CEN1B, CEN2B, CEN3B, CEN4B, CEN6B, and CEN7B in SubB. Notably, SubB accumulated substantially more CentP-156 (81 387 copies) than SubA’ (24 118 copies). Subgenome-specific divergence was observed in distributions: SubB uniquely possessed CentP-148 repeats (24 250 copies) concentrated on chromosome 5B. Conversely, SubA’ showed exclusive retention of CentP-138 repeats, distributed across six chromosomes with the exception of CEN7A’. Intriguingly, four SubA’ centromeres (CEN2A’, CEN3A’, CEN5A’, and CEN6A) exhibited dual composition containing both CentP-138 and CentP-156 variants (Fig. 3C; Table S7).

Phylogenetic analysis revealed that the CentP satellite repeats within the genome of *P. purpureum* ‘Purple’ were divided into two distinct subclades (Fig. S11). CentP satellite repeats from the same centromeres exhibited a higher degree of sequence identity, with only a minority of CentP satellite repeats from SubA’ and SubB observed to intermingle (Fig. S11). Compared to both CentP-138 and CentP-148, the CentP-156 satellite repeats showed a higher degree of sequence divergence (Figs 3D and S12). Furthermore, CentP-156 satellite repeats within SubA’ showed higher sequence variation than those in SubB (Fig. 3D). Despite its exclusive localization to CEN5B, CentP-148 diverged into two separate clades (Fig. S11). To confirm the distinct centromeric satellite variants, we designed variant-specific probes, and we utilized these centromere variant probes in conjunction with subgenome-specific abundant LTR-RT probes for dual-color FISH identification. We observed that these variant probes produced clear and bright FISH signals at the subgenomic or chromosome-specific centromeres (Fig. 3E).

A hallmark of centromeric satellite repeat evolution is their expansion through tandem duplication, resulting in the formation of higher-order repeat (HOR) arrays—a conserved feature observed in both plant and animal centromeres [1, 21, 22]. To investigate this phenomenon in our assembly, we systematically analyzed CentP HOR organization and identified 36 026 HORs across the centromeric regions of the Purple-CEN genome (Fig. 3F; Table S8). Notably, 35.79% of CentP monomers were incorporated into larger HOR units, indicating widespread tandem duplication events (Fig. 3F; Table S8). Furthermore, the size distribution of HOR blocks exhibited high variability, following a negative exponential trend, with the largest identified block comprising 24 tandem CentP-156 monomers (3744 bp) (Fig. 3F; Table S8). Spatial analysis revealed that HORs are dispersed throughout centromeric regions, yet a substantial fraction (26%) are tightly clustered, with inter-HOR distances <100 kb (Fig. 3G; Table S9). The mean spacing between adjacent HORs was 2 kb, with a maximum separation of 7145 kb, suggesting a mosaic arrangement of dense HOR arrays interspersed with extended monomeric satellite regions (Fig. 3G; Table S9).

Similar to CentP monomers, CentP HORs showed chromosome-specific distribution (Fig. 3H). In line with observations in species such as *Homo sapiens* and *Arabidopsis thaliana* [21, 22], CentP HORs within the same centromere assembled into an array with a high degree of sequence conservation, despite their significant divergence across various centromeres (Fig. 3I). This conservation suggested a common evolutionary mechanism or functional necessity across different species for maintaining HOR array integrity. Moreover, we observed that HORs in closer physical proximity exhibit reduced variation (Fig. 3J; Figs S12 and S13), aligning with the hypothesis that satellite sequence homogenization is more prominently among repeats that are in closer physical proximity. Notably, we observed several arrays (e.g., the right array of Cen7B and the left array of CEN2B) showing higher variant distances and lower CENH3 enrichment (Figs 3K and S13), suggesting a correlation between CentP variability and CENH3-binding affinity. Hence, variation in CentP could lead to the loss of CENH3 binding sites, or vice versa.

Collectively, these findings demonstrated rapid divergence of centromeric satellite repeats between subgenomes in allotetraploid *P. purpureum* ‘Purple’, revealing dynamic centromere evolution following whole-genome duplication and subgenome reshuffling in allopolyploids.

### Subgenome-biased amplification of CRP in *P. purpureum* ‘Purple’

**Figure 4:**
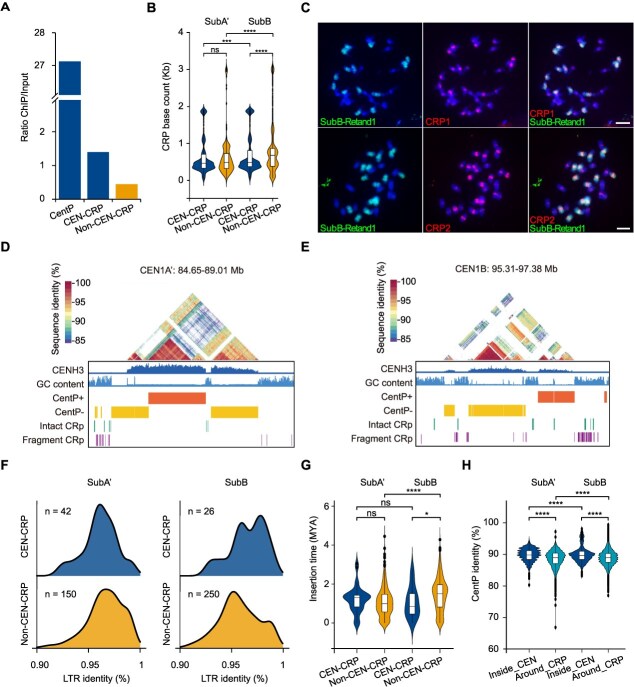
Subgenome-biased amplification of CRP in *P. purpureum* ‘Purple’. (A) CENH3 enrichment level of CentP satellite, centromeric intact CRP and non-centromeric intact CRP elements in *P. purpureum* ‘Purple’. (B) The enrichment of CEN-CRP and non-CEN-CRP elements in SubA’ and SubB. (C) FISH mapping of two CRPs in *P. purpureum* ‘Purple’. Chromosomes counterstained with DAPI. FISH signals of two CRP probes in red color, and corresponding subgenome B-specific abundant LTR-RT SubB-Retand1 in green color. Scale bar = 5 μm. (D and E) The distribution of CRPs in CEN1A’ and CEN1B. (F) LTR identity of CEN-CRPs and non-CEN-CRPs in SubA’ and SubB. (G) The insertion time of CEN-CRPs and non-CEN-CRPs in SubA’ and SubB. (H) CentP identity surrounding centromeric CRP sites and in the flanking regions in SubA’ and SubB.

In most plant species, centromeric regions are characterized by the preferential accumulation of centrophilic retrotransposons alongside satellite repeat arrays [1, 22, 29]. We re-analyzed CENH3 ChIP-seq data and observed that Centromeric Retrotransposon in *P*ennisetum (CRP) in the centromeres (CEN-CRP) were significantly less abundant than CentP satellite repeats but maintained higher levels compared to CRP outside the centromeres (non-CEN-CRP) (Fig. 4A; Table S6). Both CEN-CRP and non-CEN-CRP elements were notably enriched in SubB relative to SubA’ (Fig. 4B). Specifically, 68 intact CRP elements were detected in core centromeric regions, with 42 localized to SubA’ and 26 to SubB (Table S10). In contrast, 400 full-length non-CEN-CRP elements were identified outside centromeres, with 150 in SubA’ and 250 in SubB (Table S10). Phylogenetic analysis revealed that two CEN-CRPs (CRP1 and CRP2) formed distinct clusters with reduced diversity and shorter branch lengths, while non-CEN-CRP elements displayed higher sequence divergence (Fig. S14), suggesting postintegration duplication events for CEN-CRPs. To ascertain the variation in the abundance of CRP1 and CRP2 subfamilies between subgenomes, we conducted a dual-color FISH assay using the CRP1 and CRP2 probes in conjunction with the SubB-Retand1 probe. FISH analyses demonstrated stronger colocalization signals between CRP subfamily probes and SubB-Retand1, validating their preferential enrichment in SubB (Figs 4C and S15). Careful analysis of CRP1 and CPR2 in SubA’ and SubB further validated their significant accumulation in SubB centromeres (Figs 4D and E, S16, and  S17). Notably, we observed that CRPs embedded within CentP arrays were less abundant than the surrounding satellite sequences (Figs 4D and E, S16, and  S17), implying that high levels of CRP integration may be detrimental to centromere function in elephant grass. LTR comparisons indicated that CEN-CRPs (*n* = 68) are evolutionarily younger, with an average LTR identity of 98.7%, surpassing that of non-CEN-CRPs (96.9%, *n* = 400) (Fig. 4F). The insertion time distribution of CRPs reveals that CEN-CRP elements within the CentP array are significantly younger than non-CEN-CRP elements (Fig. 4G). Moreover, we profiled CentP variants surrounding centromeric CRP sites and observed an increase in satellite divergence in the flanking regions (Fig. 4H), suggesting that CRP insertion may impact the mutability of surrounding satellites, or retrotransposon insertions may have influenced the subsequent divergence or homogenization of adjacent CentPs.

We also observed that the preferential insertion of the centrophilic CEN-CRP families varies among different centromeres (Figs 4G, S16, and  S17), which may arise at the level of integration or differential maintenance of CRP elements within satellite CentP arrays following integration. Several large satellite array blocks (CEN2A’, CEN6A’, CEN2B, and CEN5B) are spaced far apart and are interrupted by abundant CRP invasions (Figs 1B, 4G, and S16). In both subgenomes, the majority of CRP elements exhibited fragmentation and nested insertion events (Figs 4G, S16, and  S17). However, compared to CRP elements outside of the centromere (12.7% in the external region), CEN-CRP elements constitute a minority of sequences within the CentP arrays (approximately 1.7% internally) (Figs 4G, S16, and  S17), leading us to hypothesize that internal centromeric recombination events more frequently target the elimination of CRP. Notably, the unique CRP2 insertion in CEN5A’ exhibited significantly lower CentP sequence identity and higher HOR scores in flanking satellites compared to elements shared with CEN5B (Fig. S17). This may reflect distinct spatial integration preferences of the centrophilic CRP family within the CentP arrays. The CENH3 ChIP-seq signal for CRP elements located within the CentP arrays is lower than the signal from surrounding satellites (Fig. 4A), suggesting that high levels of CRP integration may be detrimental to the centromeric function in elephant grass. These observations support a model in which a CentP homogenization pathway operates within the centromere satellite arrays to clear CRP elements in elephant grass.

In summary, the comparison of these complete assembled centromeres indicates that CRP insertions surrounding CentP exhibit a significantly higher degree of satellite DNA polymorphism in the elephant grass genome. These findings support a model where CentP homogenization pathways act to purge CRP elements from centromeric satellite arrays in elephant grass.

### Conserved epigenetic landscapes between two subgenomes despite rapid centromeric DNA evolution in *P. purpureum* ‘Purple’

**Figure 5:**
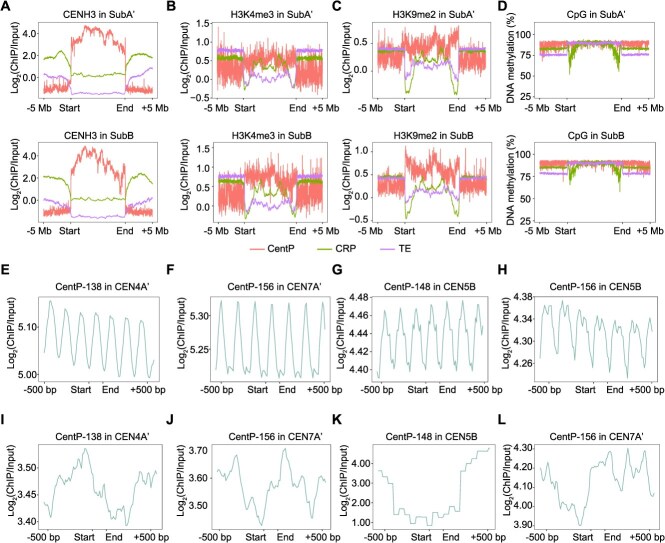
Epigenetic landscapes between two subgenomes in *P. purpureum* ‘Purple’. (A-C) Metaprofiles of CENH3, H3K4me3, and H3K9me2 ChIP-seq signals around centromeric CentP satellites and CRP retrotransposons, and CENH3 domains. (D) HiFi-derived percentage of DNA methylation in CHG contexts across three types of centromeres. (E-L) Plots of CENH3 ChIP enrichment in homogenized CentP regions (E-H) and in divergent CentP regions (I-L), averaged over windows centered on CentP-138, CentP-148, CentP-156 starts in SubA’ and SubB.

The kinetochore is a proteinaceous complex marked by the presence of nucleosomes containing the histone H3 variant CENH3, which is crucial for the segregation of chromosomes during meiosis and mitosis [1]. Since CENH3 is a hallmark of functional centromeres, we investigated the enrichment of CENH3 in CentP, CRP, and other TEs. CentP and CRP were the dominant sequences in centromeric high-enrichment regions, with CentP being more abundant (Fig. 5A), suggesting that these two classes of repetitive sequences may jointly shape the structure of the centromere. In contrast, the enrichment level of CENH3 in other TEs is significantly lower than in CentP and CRP (Fig. 5A), indicating that these sequences tend to be purged by the centromeres of *P. purpureum* ‘Purple’.

Centromeres also harbor other epigenetic marks that are essential for their functionality [1, 22, 29]. To assess the epigenetic characteristics of centromeres in *P. purpureum* ‘Purple’, we analyzed two histone ChIP-seq datasets (including the transcriptional activity mark H3K4me3 and the heterochromatic mark H3K9me2). In plants, H3K4me3 is closely associated to gene density, whereas H3K9me2 is enriched on repetitive sequences in the genome [22]. We observed similar epigenetic profiles of these two histones at the centromeres of the two subgenomes (Fig. 5B and C). The enrichment of H3K4me3 on CentP within the centromere was slightly increased, while CRP and other TEs showed a significant decrease at the edges of the centromeres (Fig. 5B), indicating that the centromere has a different chromatin state compared to the adjacent heterochromatin. Compared to the adjacent pericentromeric regions, the enrichment of H3K9me2 on CentP within the centromere was similar, while CRP and other TEs were relatively less abundant (Fig. 5C). Furthermore, through DNA methylation called from HiFi reads, we observed dense CpG methylation in CentP, CRP, and other TEs within the centromere, with a slight decrease in methylation at the edges of CRP and slightly lower CpG methylation in the pericentromeric CRP and TE (Fig. 5D).

We identified similar-sized peaks of CENH3 nucleosomes on different CentP sequences in two subgenomes (Fig. 5E–H), indicating that their DNA sequences align translationally with specific periodic nucleosomes. While CENH3 nucleosomes on homogenized CentP sequences showed regular enrichment patterns (Fig. 5E–H), those in divergent CentP regions lacked periodicity (Fig. 5I–L), indicating a high correlation between CENH3 and CentP sequence variation; this aligns with CENH3 nucleosomes potentially influencing epigenetic modifications and the divergence of satellite repeat sequences.

In summary, epigenetic landscapes are conserved between the two subgenomes despite rapid centromeric DNA evolution in *P. purpureum* ‘Purple’.

### Ongoing variations in centromeric repeat sequences across different *Pennisetum* species

To investigate the evolutionary trajectory of centromeric repetitive sequences in the genus *Pennisetum*, we selected two representative species with publicly available genomic data: *P. glaucum* (2*n* = 2*x* = 14, *x* = 7) and *P. alopecuroides* (2*n* = 2*x* = 18, *x* = 9) [30]. The diploid *P. glaucum* shares the same basic chromosome number (*x* = 7) with the allotetraploid *P. purpureum*, and its genome is classified as the A genome, which exhibits a close phylogenetic relationship with the A’ subgenome of *P. purpureum*. In contrast, *P. alopecuroides* (*x* = 9) represents a phylogenetically divergent lineage within the genus, cultivated as a perennial ornamental grass. We identified all the centromeric satellite repeats in the genomes of these two closely related species and determined their sequence conservation with the conserved CentP sequences. *P. alopecuroides* exclusively harbors CentP-156 (92 369 copies), whereas *P. glaucum* possesses two CentP variants—the predominant CentP-138 (69 346 copies) and a minor CentP-156 (3562 copies) (Fig. S18; Table S11). Notably, CentP-138 in *P. glaucum* exhibits significantly higher sequence conservation than CentP-156, whereas CentP-156 in *P. alopecuroides* displays greater sequence variation (Fig. S19). This trend mirrors our observations in *P. purpureum* ‘Purple’, where CentP-156 variants consistently exhibit higher genetic diversity, whereas CentP-138 variants are more homogeneous.

To further elucidate the evolutionary dynamics of centromeric repeats in the genus *Pennisetum*, we selected additional representative species for FISH localization, including two accessions of elephant grass (*P. purpureum* ‘Dwarf’, 2*n* = 4*x* = 28, *x* = 7; *P. purpureum* ‘Guiminyin’, 2*n* = 4*x* = 28, *x* = 7), one pearl millet (*P. glaucum* ‘Douniu’, 2*n* = 2*x* = 14, *x* = 7), and five *Pennisetum* species with *x* = 9 and various ploidy levels: *P. alopecuroides* ‘Moudry’ (2*n* = 2*x* =18, *x* = 9), *P. villosum* ‘Longistylum’ (2*n* = 3*x* =27, *x* = 9), *P. orientale* ‘Bunny’ (2*n* = 4*x* =36, *x* = 9), *P. setaceum* ‘White Ladies’ (2*n* = 5*x* =45, *x* = 9), and *Pennisetum* × *abvena* ‘Rubrum’ (2*n* = 6*x* =54, *x* = 9). CentP1 and CentP3 were co-localized with the SubA’-specific probe, while CentP2, CentP4, CentP5, and CentP6 exhibited overlapping hybridization signals to the SubB-specific probe in *P. purpureum* ‘Dwarf’ and *P. purpureum* ‘Guiminyin’ (Figs S20 and S21). Additionally, two CRPs hybridized to all centromeres of these two accessions, displaying stronger hybridization signals on SubB (Figs S22 and S23). Consequently, the signal distribution patterns of CentP variants and CRPs in these two species were similar to those observed in *P. purpureum* ‘Purple’. In *P. glaucum* ‘Douniu’, only CentP1, CentP4, and CentP5 produced hybridization signals (Fig. S24), whereas the CRP probe hybridized to centromeric regions across all chromosomes (Fig. S25). Notably, none of the CentP sequences displayed centromeric hybridization signals in five *Pennisetum* accessions with a basic chromosomal number of *x* = 9 (Figs S26–S30). In contrast, CRP signals were detected across all analyzed species, albeit restricted to partial chromosomes in certain accessions (Figs S31–S35). These collective findings indicate the occurrence of rapid centromeric sequence turnover between CentP and CRP elements in the *Pennisetum* species with *x* = 9.

In summary, the sequence divergence and localization patterns of CentPs and CRPs across different chromosome base numbers (*x* = 7 or *x* = 9) and ploidy levels in *Pennisetum* species suggest a rapid turnover of these centromeric repeats among species and across chromosomes. Our findings provide valuable insights into the evolution of centromeres within and among closely related species and the implications of allopolyploidization.

## Discussion

Elephant grass has emerged as a crucial bioenergy crop for developing sustainable and eco-friendly renewable energy solutions [16]. Although two reference genomes have been previously published [18, 23], these incomplete assemblies contained substantial gaps, particularly in centromeric and telomeric regions. In this study, we assembled a near-complete genome, Purple-CEN, using HiFi sequencing and Hi-C technology. Our assembly successfully assembled all 14 centromeres and 25 out of 28 telomeres, with only three gaps located outside the centromeres of three chromosomes. Compared to Purple-LZU, which had 1836 gaps and no telomeric sequences, Purple-CEN represents a significant improvement. Furthermore, our assembly showed a higher contig N50 length (133.50 vs. 1.82 Mb) and LAI score (13.51 vs. 3.25), indicating enhanced contiguity. Our assembly also achieved higher QV (60.04 vs. 22.59), demonstrating improved base accuracy. Compared to Purple-LZU, the new assembly included an additional 1034 genes and 192.56 Mb of repetitive sequences. The near-complete reference genome, Purple-CEN, represents the highest level of completeness, reliability, and quality, and will greatly facilitate future discoveries of genomic structure and functional genes in *P. purpureum* ‘Purple’, as well as understanding the genetic and epigenetic characteristics of allopolyploid centromeres.

**Figure 6:**
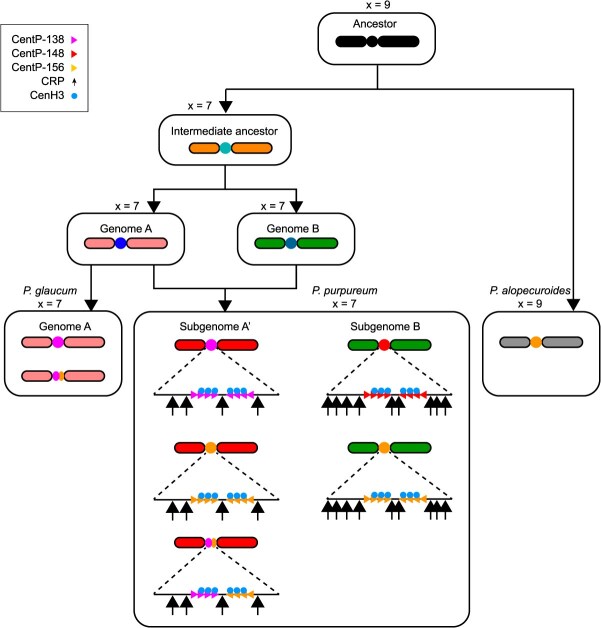
Evolution model of the dynamic centromeres in the genus *Pennisetum.* The centromeric sequences and structural conservation among *Pennisetum* species, originating from a shared ancestral foundation, underwent rapid post-speciation divergence accompanied by complete centromere turnover. Substantial differences in centromeric architecture in *P. glaucum* (2*n* = 2*x* = 14, *x* = 7) and *Pennisetum alopecuroides* (2*n* = 2*x* = 18, *x* = 9). In the tetraploid *P. purpureum* ‘Purple’ (2*n* = 4*x* = 28, *x* = 7), the fusion of centromeric repeats from its diploid progenitors has intensified subgenomic divergence of CentPs. The presence of chromosome-specific centromeric satellite clusters suggests a recombination-based homogenization mechanism operating at the chromosomal level. Notably, *P. purpureum* ‘Purple’ exhibits subgenome B-biased amplification of CRPs. Despite these genomic rearrangements, CENH3 nucleosome positioning remains relatively stable, and epigenetic homeostasis of centromeres is maintained in the tetraploid *P. purpureum* ‘Purple’.

In the vast majority of eukaryotes, satellite repeats represent the most abundant tandem repetitive sequences in centromeric regions. Their monomer length typically ranges from 150 to 180 bp, which is approximately the length of DNA wrapped around a single nucleosome [31, 32]. This pattern has been observed across a variety of monocot species, as exemplified by So1 (137 bp) in sugarcane (*Saccharum officinarum*), CentC (156 bp) in maize (*Zea mays*), CentO (155 bp) in rice (*Oryza sativa*), and both CentBd (156 bp) and CentBs (157 bp) in *Brachypodium hybridum* [14, 19, 20, 28]. Moreover, in both humans and rice, centromeric satellite repeats are highly phased with CENH3 nucleosomes, adhering to the periodicity of ‘one repeat monomer per CENH3 nucleosome’ for the stability of CENH3 nucleosomes [33]. In this study, we identified centromeric satellite repeats (CentP) in the two subgenomes of *P. purpureum* ‘Purple’, with dominant monomer lengths of 138, 148, and 156 bp (Fig. 3A). These repeats also displayed pronounced phasing pattern with CENH3 nucleosomes (Fig. 5E–H). Given their broad distribution and conservation across *Pennisetum* species, CentP repeats appear to have evolved into a stable, well-adapted component of centromeres in this genus. Nevertheless, several natural satellite-free centromeres have been documented across various species, highlighting that satellite sequences are not crucial for centromere functionality [34–37]. It was hypothesized that such repeat-free centromeres may eventually be colonized by satellite repeats, transforming into classical satellite-repeat-based centromeres [34, 38]. Notably, in potato (*Solanum tuberosum*) and chicken, the monomer lengths of newly emerged centromeric repeats vary widely, ranging from several hundred base pairs to several kilobases [34, 36]. These repeats appear to be amplified from retrotransposon-related sequences [34, 35]. Additionally, in sugarcane, three centromeric tandem repeat sequences with the monomer lengths ranging from 327 to 1371 bp are highly similar to retrotransposons, suggesting that they originated from mobile elements [28]. Consequently, retrotransposon-related sequences are a common seeding source for the origin of centromeric satellite repeats. However, these novel centromeric satellites originating from TEs lack translational phasing on CENH3 nucleosomes. Therefore, we conclude that the regular positioning of CENH3 nucleosomes represents an adaptive evolutionary feature of centromeric satellite repeats. This phasing is facilitated by monomer lengths that correspond to the amount of DNA wrapped around a single nucleosome. A defining characteristic of centromeric satellite sequences is their rapid evolution, notwithstanding the high functional conservation of centromeres [2, 22]. Typically, centromeric satellite sequences exhibit pronounced species-specificity, with remarkably low similarity observed across different species, even among chromosomes within the same species [28, 32, 34, 35]. In the centromeres of *P. purpureum* ‘Purple’, CentP sequences display extensive intrachromosomal variation, with most monomers being chromosome-specific (Figs 3E and S11), supporting a model wherein satellite repeat homogenization occurs predominantly within chromosomes [22]. A recurrent organizational pattern is observed wherein highly homogenized repeats are localized to the core centromeric region, while more divergent repeats occupy the flanking domains (Fig. 4F), consistent with previous reports in humans, *A. thaliana*, and *Brachypodium distachyon* [14, 21, 22]. Furthermore, the negative correlation between CentP divergence and CENH3 occupancy suggests that centromeric chromatin may facilitate recombination pathways leading to homogenization (Figs 1B, 3J, 3K, S12, and  S13). The presence of CentP satellite repeats across diverse *Pennisetum* species indicates their potential origin from a common ancestor (Fig. 6). Through sequence variation and comparative FISH analysis, we determined that centromeric satellite repeats have undergone differentiation across evolutionary timescales at the species, subgenome, and chromosomal levels (Figs 3B, D, and  E, S11, and  S20–S35). Therefore, it can be inferred that during evolution, multiple centromeric repeats may emerge and undergo dynamic amplification and adaptation, especially following allopolyploidization events that combine two ancestral genomes potentially containing different centromeric repeats (Fig. 6). However, in this dynamic process, the best-adapted satellite repeat will survive and eventually occupy different centromeres (Fig. 6). Similar to CentP monomer sequences, HORs also exhibit substantial chromosome specificity (Fig. 3H). Their divergence demonstrates a distance-dependent effect: repeat blocks located distally from the centromeric core display greater variation (Fig. 3J). This may be due to homogenization processes based on recombination occurring between allelic or nonallelic positions on the same chromosome, maintaining a CentP repertoire close to optimal CENH3 recruitment [22].

In addition to centromeric satellite repeats, centromeric retrotransposons constitute a significant class of TEs predominantly localized to centromeres [20, 22, 28]. The centromeres of most monocot species are predominantly enriched with Ty3-gypsy-class LTR retrotransposons, a characteristic particularly prominent among major cereal crops.

In rice, centromeres are densely populated with centromeric retrotransposons of rice (CRRs) elements, which are interspersed with CentO satellite repeats [39]. Similarly, maize centromeres are characterized by centromeric retrotransposons of maize (CRMs) elements that exhibit a comparable distribution pattern with CentC satellites [20]. Wheat centromeres harbor five distinct Ty3-gypsy centromeric retrotransposons of wheat (CRWs), with CRW1 and CRW2 constituting the predominant elements [40]. These centromeric retrotransposons are often highly abundant in monocots, underscoring their pivotal role in centromere evolution. Similar to other monocots, the centromeres of elephant grass have been invaded by CRP retrotransposons (Figs 4D and E, S16, and  S17). We detected a negative correlation between CRP density and the polymorphism of centromeric satellite arrays in elephant grass (Fig. 4H). These findings suggest that CRPs contribute to centromeric structural diversity among different chromosomes within the same subgenome and also to the differentiation of centromeric sequences and structures between subgenomes (Fig. 6). Furthermore, FISH assays confirmed the presence of these retrotransposon families in different species of the genus *Pennisetum*. However, in some *Pennisetum* species with a basic chromosome number of *x* = 9, certain chromosomes exhibit a deficiency of CRPs, and CENH3 was not yet phased to these sequences. This suggests that they may have undergone dynamic amplification and adaptation during speciation. However, notable exceptions to this common organizational pattern are found in several dicotyledonous species, revealing alternative paths of centromeric evolution. For example, in sacred lotus, centromeres are predominantly composed of Ty1-copia retrotransposons [41]. Moreover, while *Arabidopsis lyrata* centromeres are rich in the Ty3-gypsy-type Athila elements, they also harbor a substantial complement of Ty1-copia-type Ale retrotransposons, which are phylogenetically distinct and represent more recent insertions [42]. These structural differences demonstrate that the centromeric niche is not exclusive to a single class of retrotransposons. We also observed that although CRPs insert into elephant grass centromeres, they appear to be efficiently eliminated, as evidenced by the low abundance of CRP elements within the centromeres (Figs 4D and E, S16, and  S17). A similar observation was made in *Arabidopsis* centromeres, which may be due to the recombination-mediated purge of retrotransposons by centromeric satellite arrays [29]. During the evolution of centromeres, cycles of insertion and purge of LTR-RTs continuously occur. This dynamic change not only enriches the sequence composition of centromeres but may also affect their function and chromosome stability by altering the chromatin structure and epigenetic modification state. Overall, the presence of LTR-RTs in plant centromeres reveal the complexity of genome structure and provide novel insights into centromere evolution in allopolyploids. Further research will help elucidate the specific mechanisms of these LTR-RTs in centromeres and their impact on plant genome evolution.

## Conclusion

Here, we report a near-complete chromosome-resolved genome assembly of the allotetraploid *P. purpureum* ‘Purple’, achieving full resolution of all 14 centromeres. Our study uncovers that structural divergence between subgenomes originates from asymmetric expansion of six lineage-specific LTR retrotransposon families, exhibiting pronounced subgenome-biased amplification patterns. CentPs demonstrate rapid sequence divergence both between subgenomes and across individual chromosomes, whereas CENH3 histones selectively associate with conserved satellite arrays organized into HOR configurations. Notably, CRPs exhibit more recent evolutionary origins compared to non-centromeric retroelements, with striking amplification bias toward subgenome B. Moreover, CRP insertions flanking CentP satellites are linked to elevated satellite sequence variation, consistent with a model where CentP homogenization processes actively purge retrotransposon invasions from functional centromeric regions. Despite rapid sequence turnover, these centromeric regions maintain evolutionarily conserved epigenetic architectures between two subgenomes. Comparative analyses across *Pennisetum* species highlight rapid species- and chromosome-specific turnover of CentP and CRP elements, proposing their involvement in accelerating speciation. This work reveals novel insights into the adaptive landscape of allopolyploid centromeres, demonstrating how genetic innovation and epigenetic conservation jointly stabilize subgenome architecture. Our findings establish a paradigm for understanding centromere-mediated subgenome coordination in polyploid organisms, with implications for speciation dynamics and chromosomal evolution in complex genomes.

## Materials and methods

### Plant materials and genome sequencing

To generate high-quality genomic and transcriptomic datasets for *P. purpureum* ‘Purple’, plants were cultivated under controlled greenhouse conditions with a 16-hour light/8-hour dark photoperiod at a constant temperature of 25°C. High-molecular-weight DNA was extracted from young fresh leaves collected after two weeks of growth and sequenced using the PacBio Revio platform, yielding 94.83 Gb of HiFi reads for *de novo* genome assembly. To further enhance chromosomal scaffolding, Hi-C libraries were prepared from the leaf tissue and sequenced on the Illumina NovaSeq 6000 platform, generating 207.41 Gb of chromatin interaction data. Additionally, RNA-seq was performed on leaves, roots, and stems using the same Illumina platform to support transcriptome-assisted gene prediction and annotation.

### Genome assembly

Prior to genome assembly, genome survey was conducted. We analyzed the HiFi sequencing data using Jellyfish (v2.3.0, https://github.com/gmarcais/Jellyfish) for *k*-mer (*k* = 21) statistics. The *k*-mer distribution was visualized using ggplot2 (v3.5.1, https://github.com/tidyverse/ggplot2). Genome properties such as haploid genome size, heterozygosity, and repeat content were estimated using GCE (v1.0.2, https://github.com/fanagislab/GCE). Genome assembly was performed using hifiasm (v0.19.9-r616, https://github.com/chhylp123/hifiasm), which assembled the HiFi reads into contigs. To scaffold these contigs, the HapHiC (v1.0.6, https://github.com/zengxiaofei/HapHiC) pipeline was employed. Hi-C data were aligned to the contig sequence using BWA (v.0.7.17) [43]. After quality control and filtering, the contigs were manually adjusted using Juicebox (v2.20.00, https://github.com/aidenlab/Juicebox). The 3D-DNA (v201008, https://github.com/aidenlab/3d-dna) pipeline was then used to construct chromosomes. To close gaps, additional contig assemblies were generated from HiFi data using HiCanu (v2.2) and NextDenovo (v2.5.2) [24, 25]. quarTeT (v1.2.1) was used to fill the gaps within the chromosomes [44]. Finally, the assembled genome was polished using NextPolish2 (v2.0.2.1, https://github.com/Nextomics/NextPolish2) with HiFi and NGS data, yielding the final genome assembly, named Purple-CEN [45].

### Validation of the genome assembly

The previously published genome (Purple-LZU) [18] was aligned to the Purple-CEN genome using MUMmer (v4.0.0rc1, https://github.com/mummer4/mummer) [46]. The alignment and comparison of structural variations were conducted using nucmer with the following parameters: ‘nucmer -g 1000 -c 90 -l 40’, ‘delta-filter -r -q -l 1000’, and ‘show-coords -TrHcl’. The genome feature was visualized using GenomeSyn (v1.2.7, https://github.com/jmsong2/GenomeSyn) [47]. SubPhaser (v1.2.6) was employed to analyze the sequence characteristics of subgenomes [26]. For collinearity analysis, the coding DNA sequences from other genomes were aligned to the Purple-CEN genome using LAST (v1.04.15) [48]. Collinear blocks were identified using JCVI (v1.4.16, https://github.com/tanghaibao/jcvi), and collinearity dot plot was generated [49].

To assess genome quality, several metrics were evaluated. QV were estimated using Merqury (v1.3, https://github.com/marbl/merqury). Genome completeness was assessed using BUSCO (v5.0.0, https://gitlab.com/ezlab/busco) with the Embryophyta_odb10 gene set. The LAI was evaluated using LTR-retriever (v3.0.1, https://github.com/oushujun/LTR_retriever). Gaps and telomere structures were assessed using quarTeT [44].

### Gene model and repeat sequence annotation

To annotate the gene model, RNA-seq data from root, stem, and leaf tissues were aligned to the Purple-CEN genome using HISAT2 (v2.2.1) [50]. Transcripts were subsequently assembled and annotated with StringTie (v2.2.3). Protein sequences from four closely species (*O. sativa*, *Z. mays*, *Sorghum bicolor*, and *Oil palm*) were collected, and redundant sequences were removed using CD-HIT (v4.8.1, https://github.com/weizhongli/cdhit) to create a non-redundant protein database [51]. Gene prediction was performed using BRAKER3 (v3.0.8, https://github.com/Gaius-Augustus/BRAKER) and PASA (v2.5.3, https://github.com/PASApipeline/PASApipeline) was employed to obtain high-quality protein-coding genes with default parameters [52]. Functional annotation of protein-coding genes was conducted using multiple databases, including EggNOG-mapper (v2.1.12, https://github.com/eggnogdb/eggnog-mapper) with the EggNOG (v5.0.2) dataset [53]. InterProScan (v5.65–97.0) was applied to annotate gene functions through the InterPro database. KEGG annotations were performed using Kofam (v1.3.0, https://www.genome.jp/ftp/tools/kofam_scan/) [54]. Finally, DIAMOND (v2.1.9.163, https://github.com/bbuchfink/diamond) was used to align protein sequences to the NR and SwissProt databases for functional annotation [55]. Repetitive sequences in the Purple-CEN genome were predicted using RepeatModeler (v2.0.5, https://github.com/Dfam-consortium/RepeatModeler) to create a non-redundant repeat library [56]. Repetitive element annotations were performed using EDTA (v2.2.0, https://github.com/oushujun/EDTA), RepeatMasker (v4.1.7-p1, https://github.com/Dfam-consortium/RepeatMasker), and TEsorter (v1.4.6, https://github.com/zhangrengang/TEsorter) [57, 58].

### ChIP-seq and data processing

Chromatin immunoprecipitation (ChIP) was performed on young leaves using anti-CENH3 antibody from rice [39], following a previously established protocol [28]. The ChIP libraries were prepared using the NEBNext®Ultra™ DNA Library Prep Kit (New England BioLabs Inc., Ipswich, MA, USA) and sequenced on Illumina NovaSeq 6000 platform, producing 150 bp paired-end reads. ChIP-seq with antibodies against H3K4me3 (rabbit, Abcam Ab8580) and H3K9me2 (mouse, Abcam Ab1220) was performed as mentioned above. Paired-end reads were processed using fastp (v0.23.4) to filter low-quality bases and aligned to the Purple-CEN genome with bowtie2 (v2.4.4) using the parameters: –very-sensitive, −no-mixed, −no-discordant, −k 10. Reads with a mapping quality below 10 and duplicate reads were removed using samtools (v1.20).

### CpG methylation analysis

The methylation probability of each CpG site within HiFi reads was estimated using Primrose v1.3.0 (https://github.com/PacificBiosciences/primrose) with default parameters. The alignment of the reads was performed using pbmm2 v1.9.0 (https://github.com/PacificBiosciences/pbmm2). The percentage of methylation at each genomic position was calculated using pb-CpG-tools (https://github.com/PacificBiosciences/pb-CpG-tools) with the parameter -p model.

### Identification and characterization of centromeric repeat sequence

ChIP-seq and input reads were trimmed using trim_galore (v0.6.4_dev) (https://www.bioinformatics.babraham.ac.uk/projects/trim_galore) and aligned to the reference genome with Bowtie2 (v.2.3.4.1). The aligned reads were then filtered with SAMtools (v.1.9) to retain only properly paired reads with a mapping quality score ≥ 10. CENH3-associated domains (i.e., centromeric regions) were identified by comparing the ChIP samples to the input controls using MACS2 (v2.2.9.1) with the following parameters: callpeak -t ChIP.bam -c Input.bam -f BAM -g 1.3e9 -n CENH3 -B -q 0.05—broad. Tandem Repeat Analyzer (TAR) (v4.09.1) was used to detect satellite monomer sequences on individual chromosomes. The number, variation, and sequence identity of satellite monomers were determined using LASTZ (v1.04.41). Centromeric satellite repeats were visualized using the StainedGlass (v0.6) [59], and higher order repeat (HOR) arrays within each chromosome were identified with HiCAT (v1.1.0) [60]. Histone modification and CENH3 enrichment analyses were conducted using the computeMatrix tool in deepTools (v3.5.5) in ‘scale-regions’ mode, with parameters: -regionBodyLength 2000 -beforeRegionStartLength 2000-afterRegionStartLength 2000.

### Phylogenetic tree of centromeric repeats

The CentP satellite repeats were extracted using LASTZ (http://www.bx.psu.edu/~rsharris/lastz/) for the Purple-CEN genome, and were used for further phylogenetic analysis. CRP elements were extracted from the Purple-CEN genome and divided into locations inside or outside the core centromeres. 1000 randomly selected sequences were aligned using MAFFT (v7.525) with default parameters [61]. Phylogenetic tree was constructed using FastTree (v2.1.11) and visualized using iTOL (https://itol.embl.de/) [62, 63].

### Genome-wide comparative analysis of LTR-RT abundance in in *P. purpureum* ‘Purple’

The genome of *P. purpureum* ‘Purple’ was computationally fragmented into 100-nt sequences, and subgenome-specific LTR-RT abundance ratios were quantified using RepeatExplorer2, a Galaxy-based platform for eukaryotic repeat characterization via graph-based sequence similarity clustering [64]. Bidirectional pairwise comparisons between subgenomes (SubA’/SubB and SubB/SubA’) were performed to identify subgenome-enriched LTR-RT families, with candidates selected under a stringent enrichment threshold (subgenomic proportion ratio > 10). This analysis resolved two SubA’-enriched and four SubB-enriched LTR-RT subfamilies. Putative subgenome-specific LTR-RTs were subjected to BLASTN alignment (*E*-value ≤1e−5) against the EDTA-curated TE database of *P. purpureum* ‘Purple’. Sequences demonstrating both nucleotide identity and alignment coverage ≥90% were retained as validated elements.

### Chromosome immunofluorescence and fluorescence *in situ* hybridization

Chromosome immunofluorescence and fluorescence *in situ* hybridization (FISH) experiments were conducted following the methods of Huang et al [28]. For immunostaining, a CENH3 antibody developed in rice was used [39]. For the FISH experiment, the probes were amplified by PCR using sequence-specific primers (Table S12), followed by enzymatic labeling with either digoxigenin-11-dUTP (Roche Diagnostics, Basel, Switzerland) or Biotin-16-dUTP (Roche Diagnostics, Basel, Switzerland) via nick translation. The hybridization mixture (10 μl/slide) contained 50% deionized formamide, 10% dextran sulfate, 2× SSC (pH 7.0), 50 ng of each labeled probe. Slides were denatured at 75°C for 3 min on a thermal cycler prior to overnight hybridization at 37°C in a humidified chamber. After hybridization, the slides were subjected to standard stringent washes. Following FISH, the hybridization signals were detected using Alexa Fluor 488 streptavidin (Thermo Fisher Scientific, Waltham, MA, USA) and rhodamine-conjugated anti-digoxigenin antibody (Roche Diagnostics, Basel, Switzerland). The chromosomes were then counterstained with a DAPI in a Vectashield antifade solution (Vector Laboratories Inc., Burlingame, CA, USA). The chromosomes and FISH signals were observed using an Olympus BX53 fluorescence microscope (Olympus, Tokyo, Japan), and images were captured using cellSens Dimension v.1.9. Finally, image contrast was adjusted using Adobe Photoshop CC (https://www.adobe.com).

## Supplementary Material

Web_Material_uhaf301

## Data Availability

The whole-genome sequencing data (including Illumina short reads, HiFi reads, and Hi-C interaction reads), ChIP-seq data, and transcriptomes of different tissues used in this study have been deposited at the National Genomics Data Center (NGDC) under accession number PRJCA034549. The raw reads used in this study are available under the following accession numbers (ChIP-seq data: CRA021877; RNA-seq data: CRA021875; Illumina WGS data: CRA021874; HiFi data: CRA021873; Hi-C data: CRA021871). The genome assembly and gene annotation data for *P. purpureum* ‘Purple’ have been deposited at the Genome Warehouse (GWH) under accession number GWHFIJS00000000.1.
