# Supplementary material for: The near-complete genome assembly of allotetraploid Pennisetum purpureum ‘Purple’ reveals the genetic and epigenetic landscape of centromeres
Source: Hortic Res. 2025 Oct 29;13(2):uhaf301. doi: 10.1093/hr/uhaf301 (PMC12933667; doi:10.1093/hr/uhaf301)
Supplement: Web_Material_uhaf301 [file web_material_uhaf301.zip › Supplementary Figure.pdf]

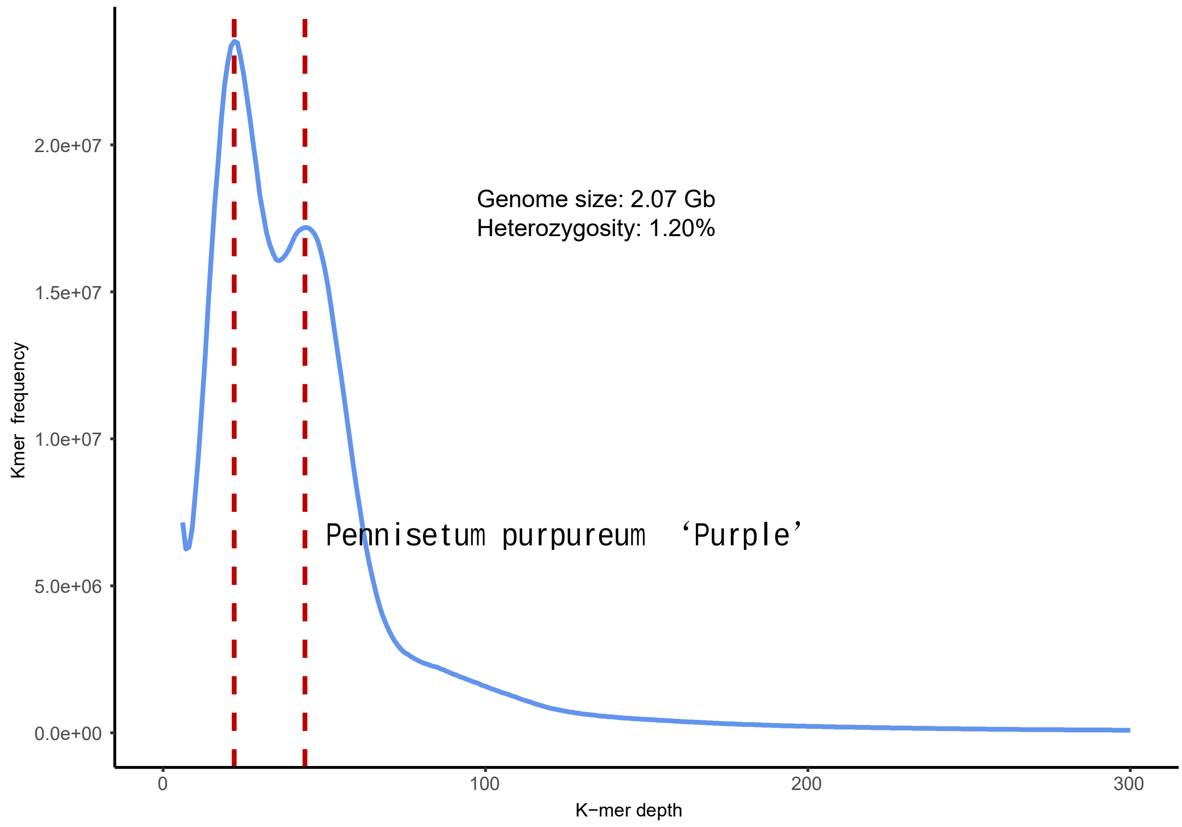

Fig. S1 K-mer analysis of *Pennisetum purpureum* 'Purple' with GCE (k=17).

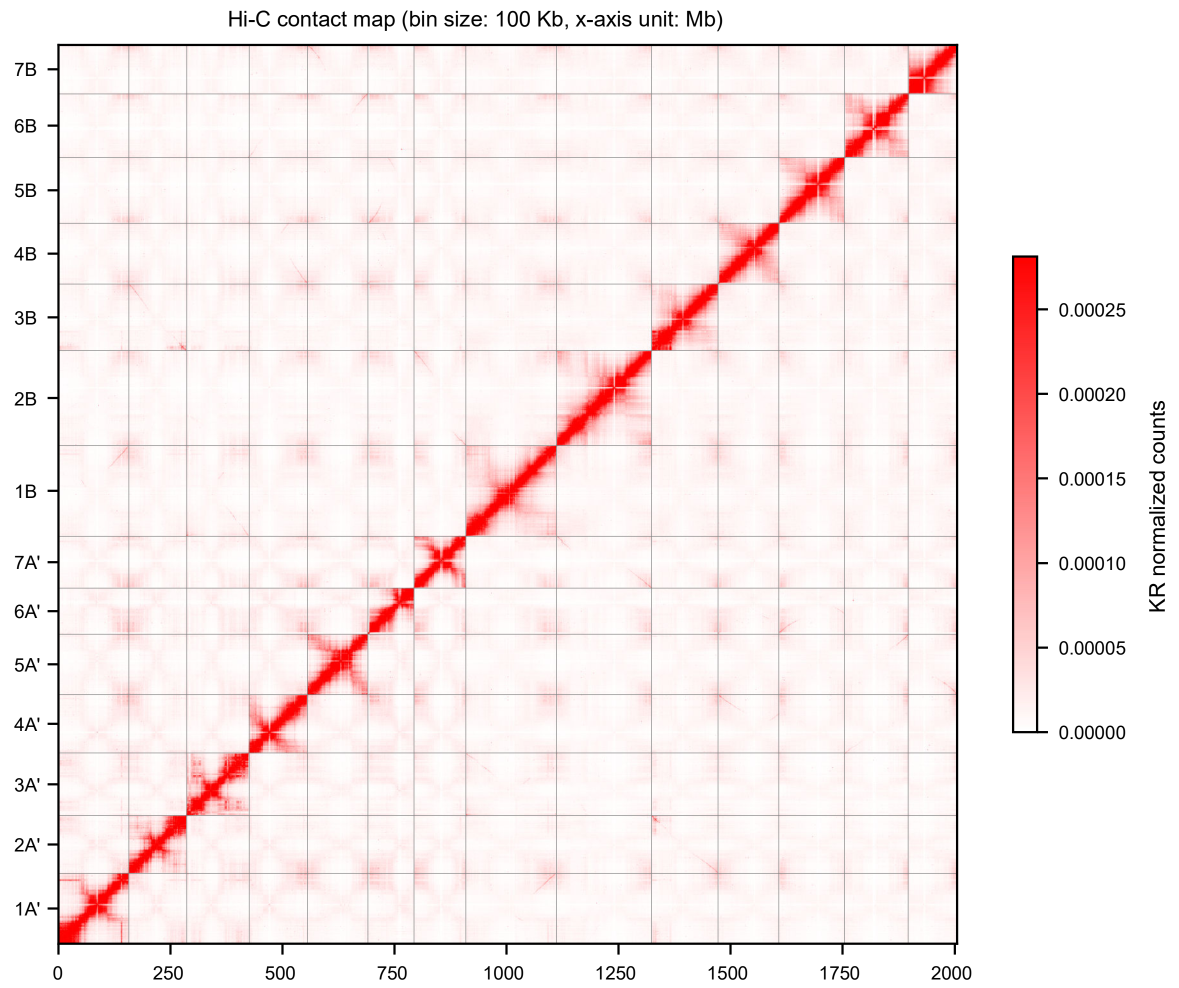

Fig. S2 Genome-wide chromatin interaction in Purple-CEN genome assembly at 100-Kb resolution. The intensity of pixels represents the links between 100-kb windows on all chromosomes. Darker red color indicates a higher contact probability, and the white space represents little or no contacts.

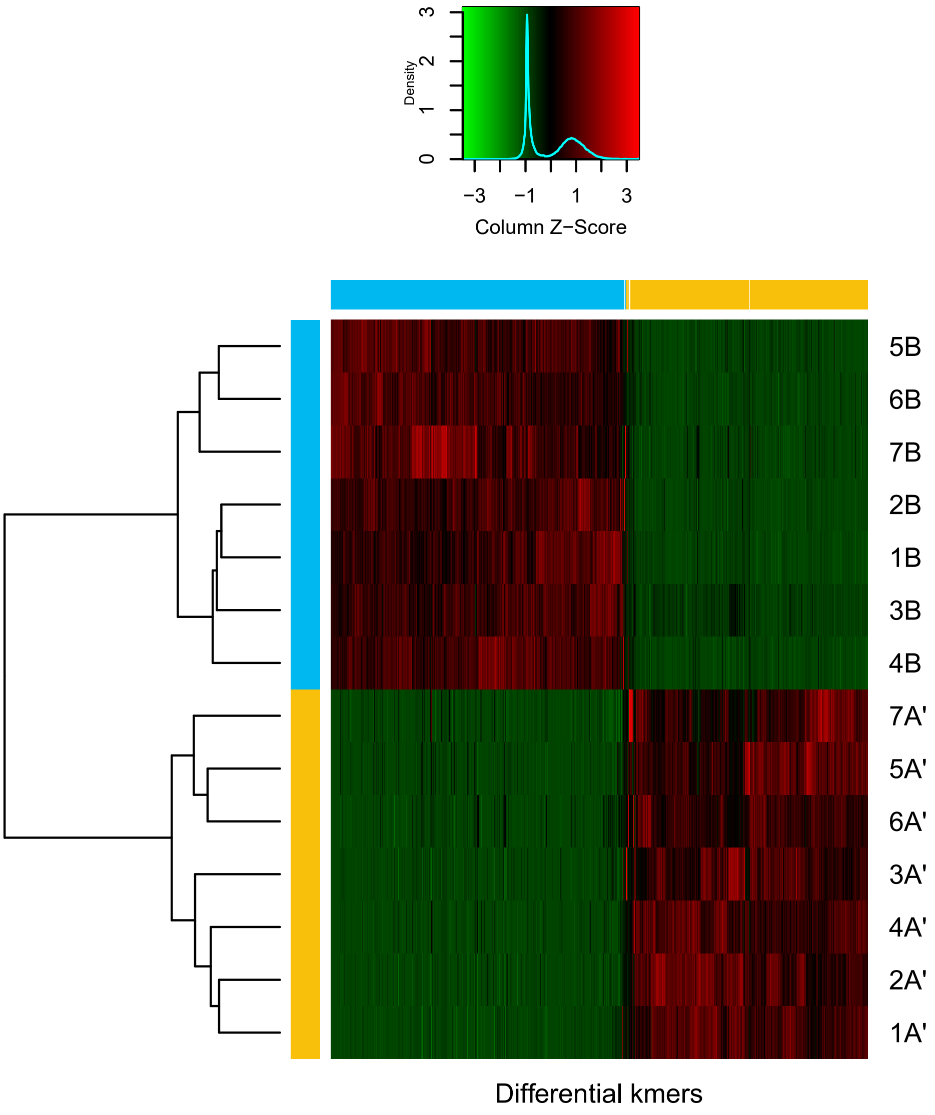

Fig. S3 Distinguishment of subgenomes based on subgenome-specific k-mers. Unsupervised hierarchical clustering (the horizontal color bar at the top of the axis indicates to which subgenome the k-mer is specific; the vertical color bar on the left of axis indicates the subgenome to which the chromosome is assigned. The heat map indicates the Z-scaled relative abundance of the k-mers. The larger the Z score, the higher the relative abundance of a k-mer).

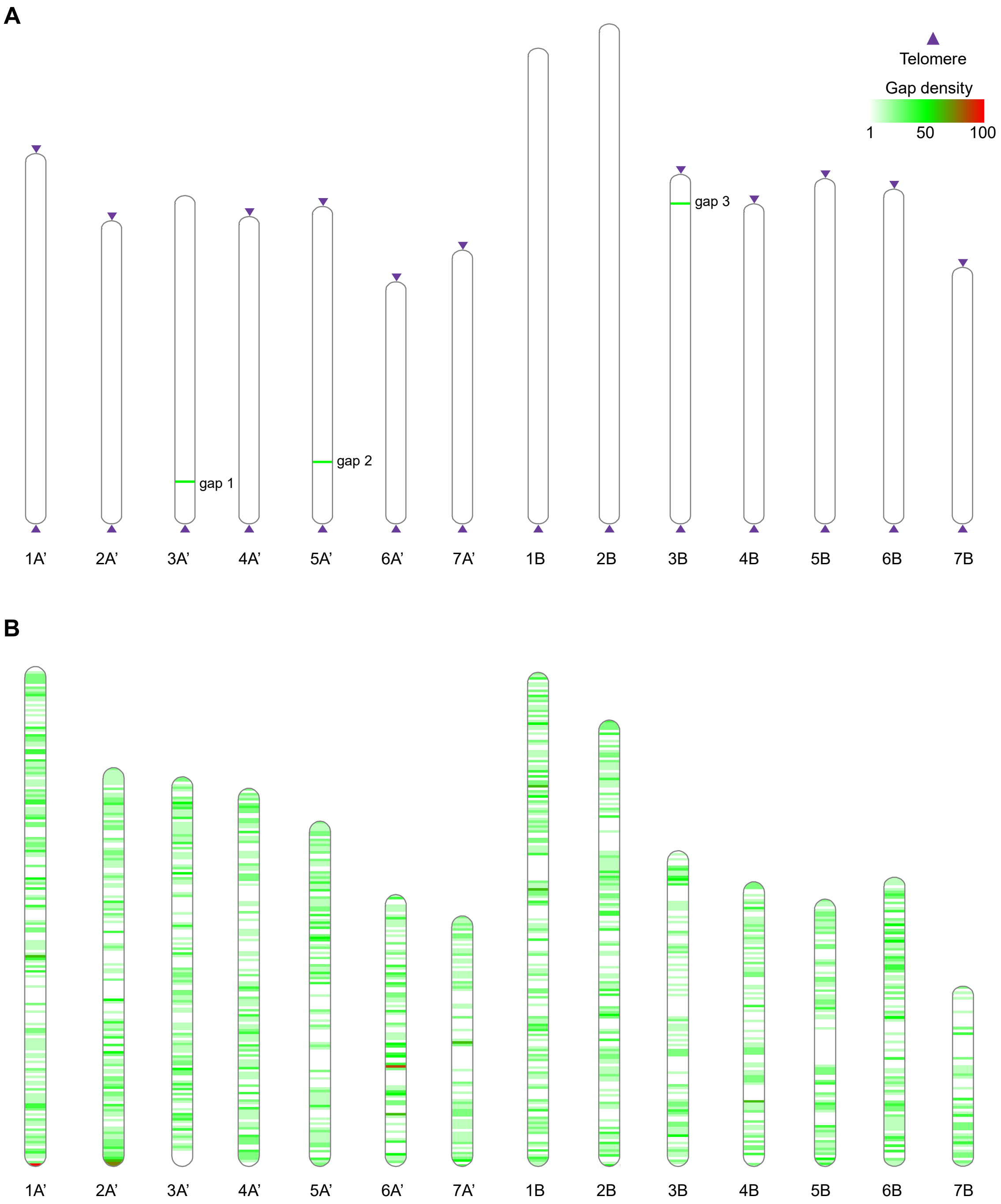

Fig. S4 Chromosome distribution of telomeres and gaps in Purple-CEN (A) and Purple-LZU (B).

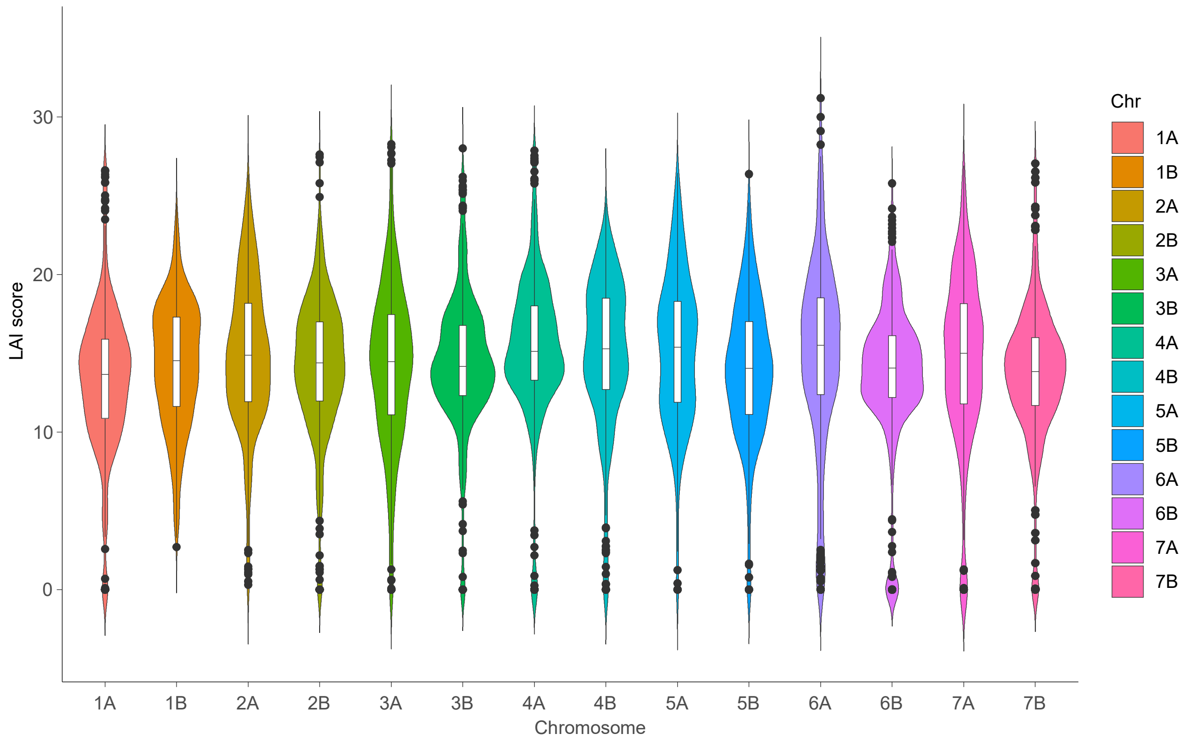

Fig. S5 LAI score of 14 chromosomes in Purple-CEN genome assembly.

A

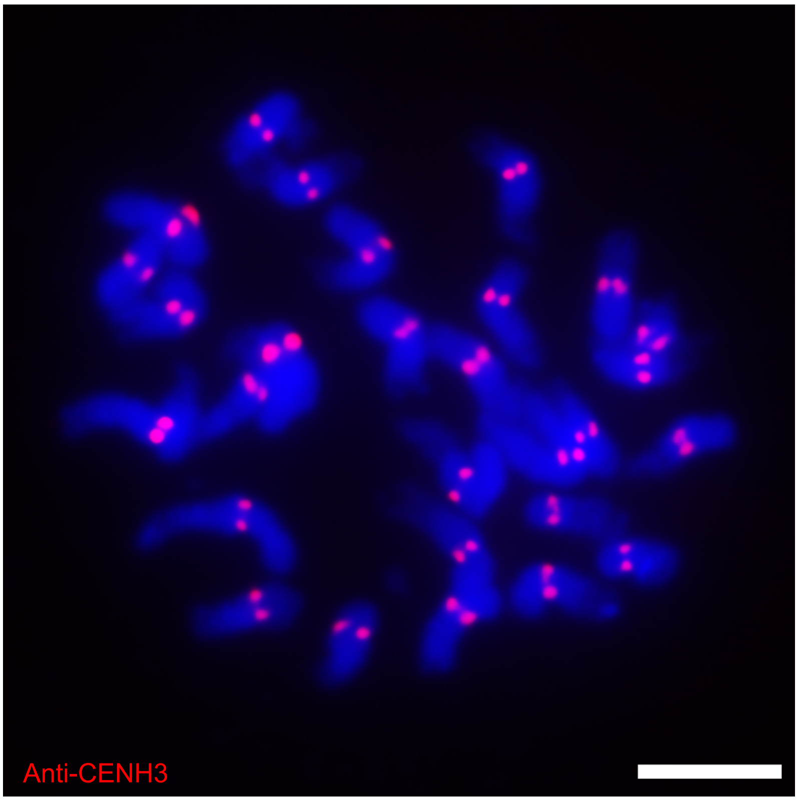

B

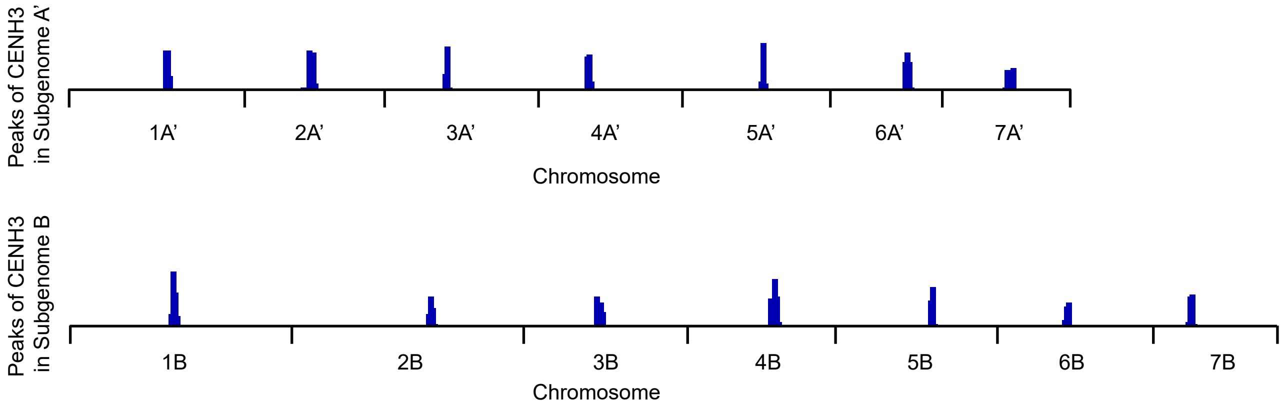

Fig. S6 Immunostaining signals of anti-CENH3 antibody and identification of CENH3 binding region on each chromosome of *Pennisetum purpureum* 'Purple'.

(A) The immunostaining signals of anti-CENH3 antibody are colored in red. Chromosomes counterstained with DAPI are in blue. Scale bar = 5 μm. (B) The blue histogram represents the normalized read count (RPKM normalization) ratio between ChIP-seq reads and input reads.

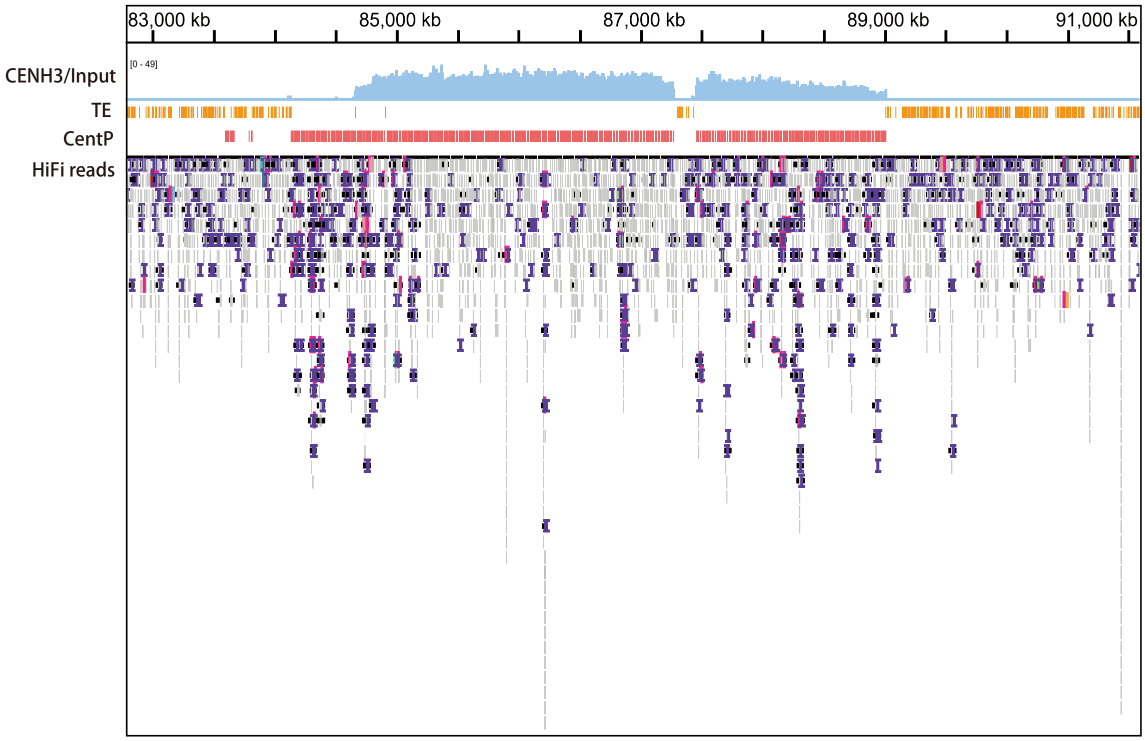

Fig. S7 IGV screenshots in the centromere regions of Purple-CEN.

The top track shows the density of TE, quantified as the number of transposons per kilobase (1-kb). Upon integration into the IGV platform for heatmap scale normalization, data values equal to or below -1.5 are represented in a deep blue, whereas values equal to or surpassing 1.5 are depicted in a deep red. Values spanning from -0.1 to 0.1 are correspondingly mapped onto a white color spectrum, providing a nuanced visualization of the genomic data. The middle track displays the coverage of PacBio HiFi reads. The alignment of PacBio HiFi reads (lower track) is shown. The purple marks indicate insertion.

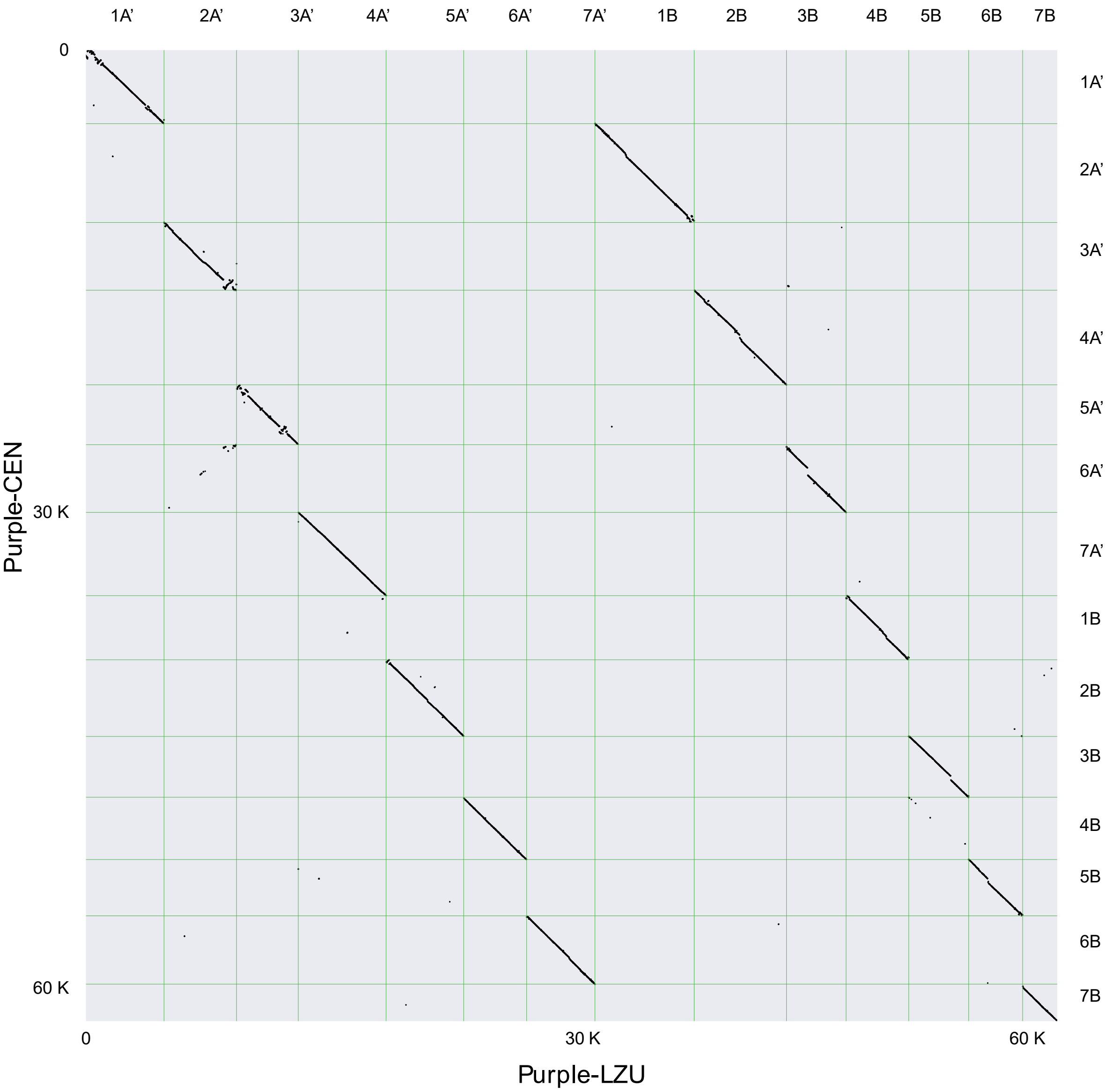

Fig. S8 Inter-genomic comparison between Purple-CEN and Purple-LZU based on pairs of collinear genes (97,038 gene pairs).

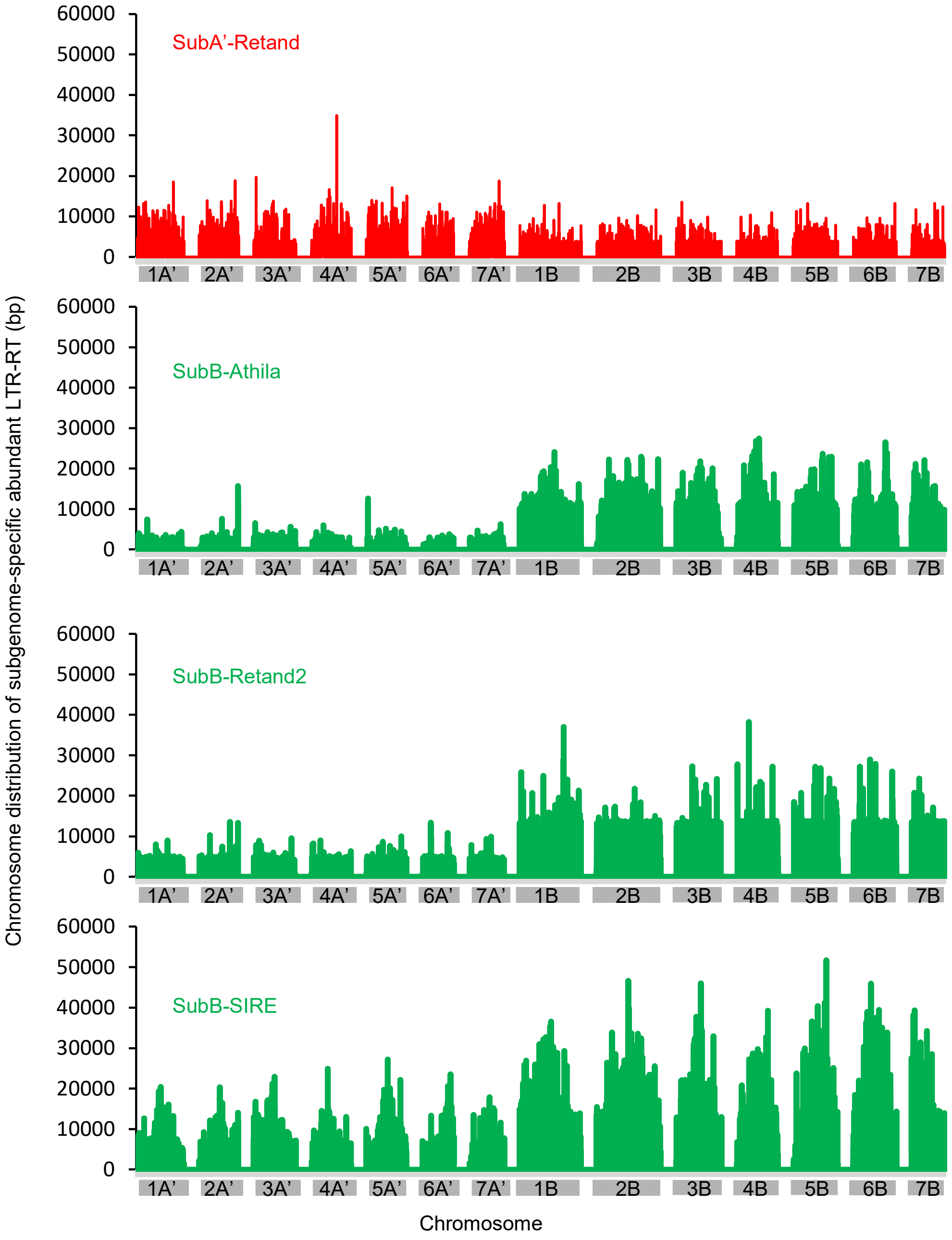

Fig. S9 Chromosome distribution of subgenome-specific abundant LTR-RTs. in the subgenomes of *P. purpureum* ‘Purple’.

SubA’-Retand in red color, and SubB-Athila, SubB-Retand2, and SubB-SIRE in green color, respectively.

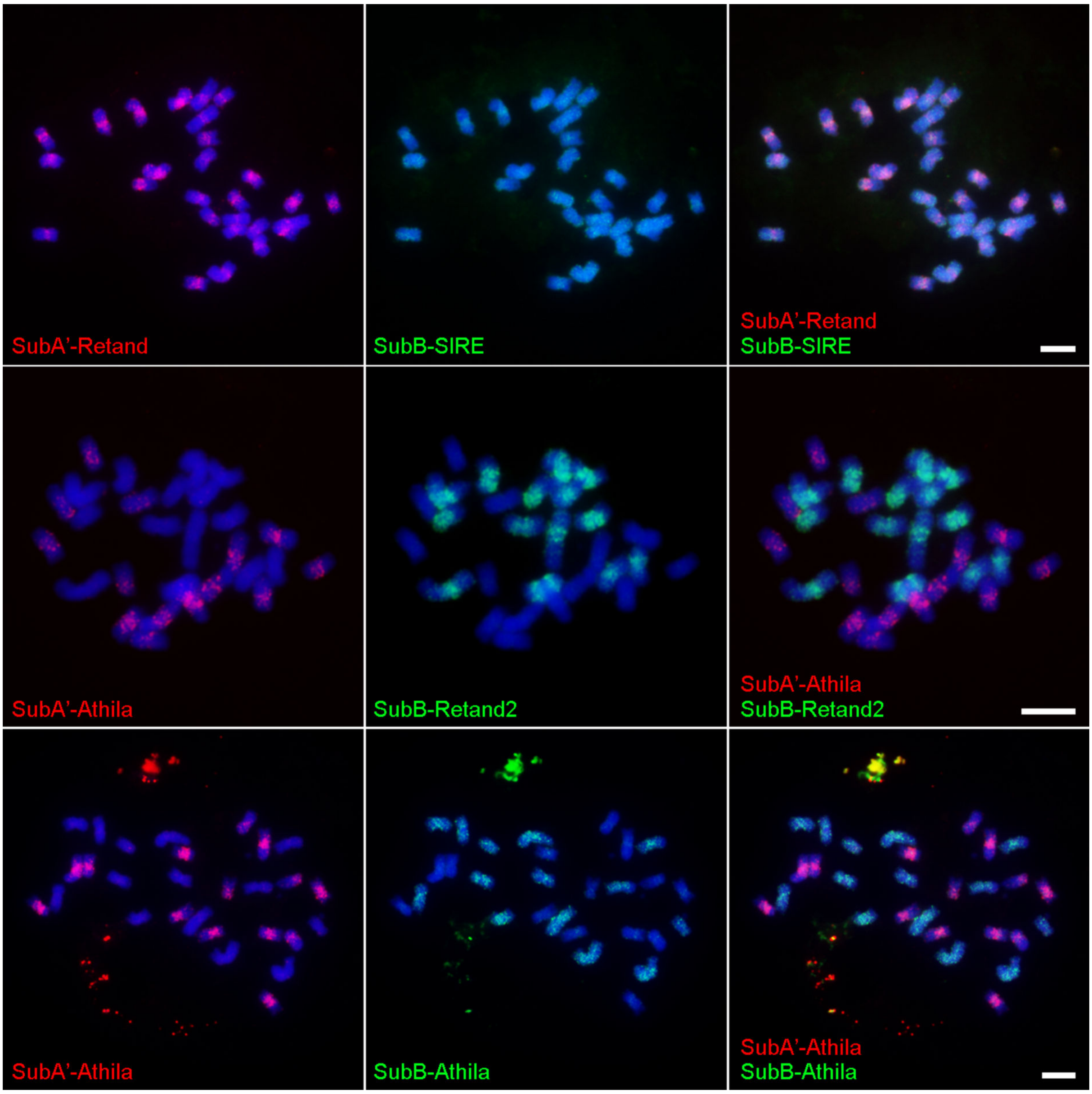

Fig. S10 FISH mapping of two subgenome-specific abundant LTR-RTs in *P. purpureum* 'Purple'. Chromosomes counterstained with DAPI. FISH signals of probes SubA'-Athila (red), SubA'-Retand (red), SubB-SIRE (green), SubB-Retand2 (green), and SubB-Athila (green), respectively. Scale bar = 5 μm.

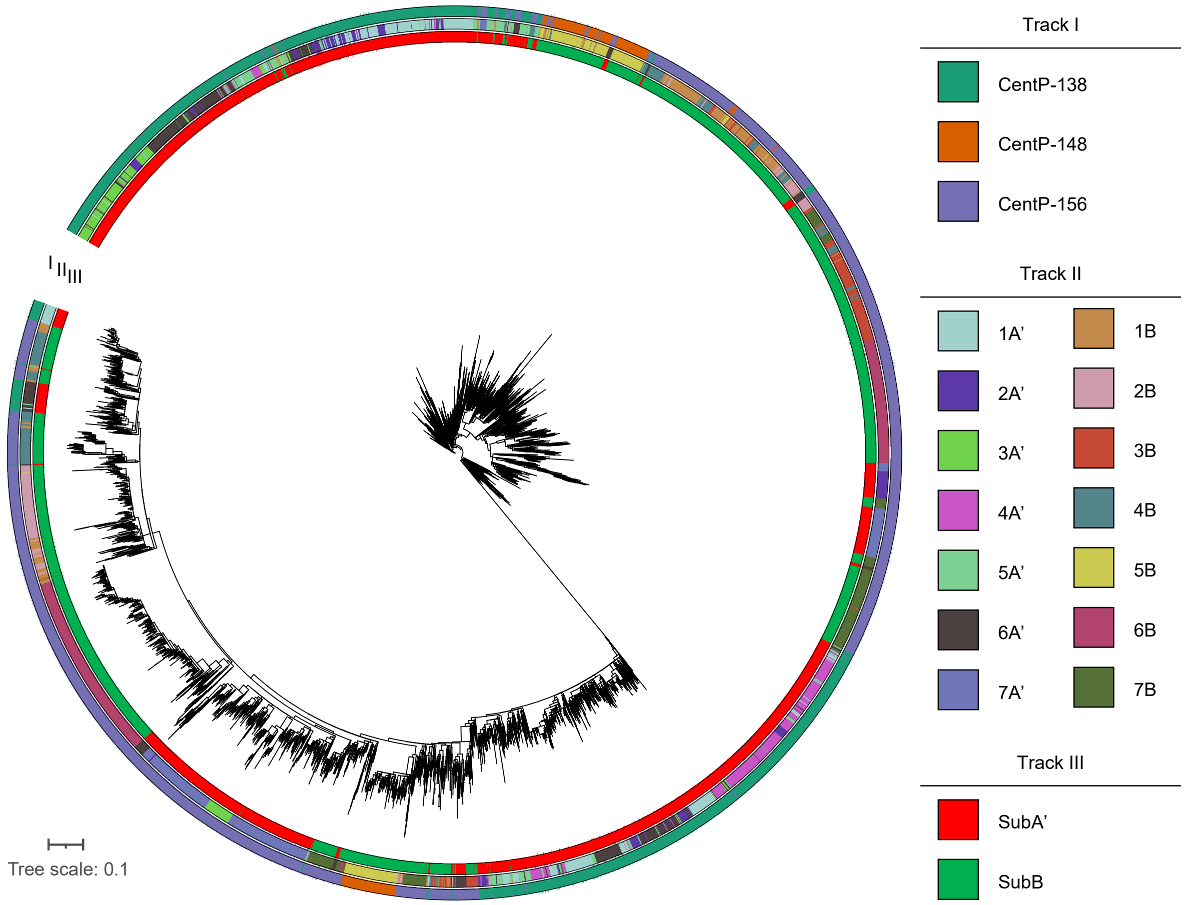

Fig. S11 Phylogenetic tree of CentPs in the centromeres in the subgenomes of *P. purpureum* 'Purple'.

Phylogenetic tree for CentP-138 (green), CentP-148 (orange), CentP-156 (purple) in the SubA' (red) and SubB (green) of *P. purpureum* 'Purple'.

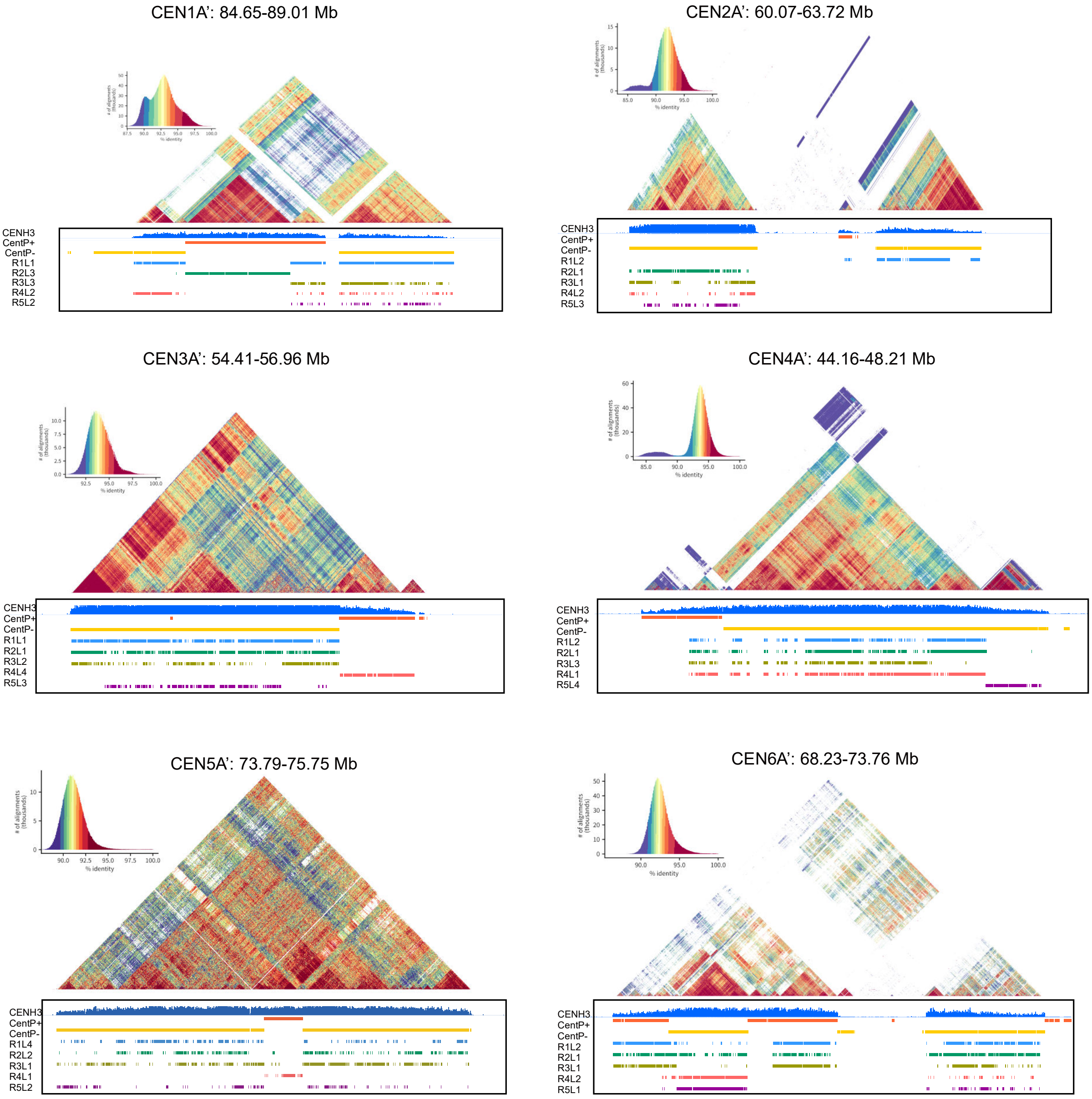

Fig. S12 Higher order structure analysis of CentP satellite DNA arrays in the subgenome A of *P. purpureum* 'Purple'.
StainedGlass sequence identity heatmap of putative centromeric regions of the subgenome A. Histograms at the top left show the assignment of colors to sequence identity values for each heatmap.

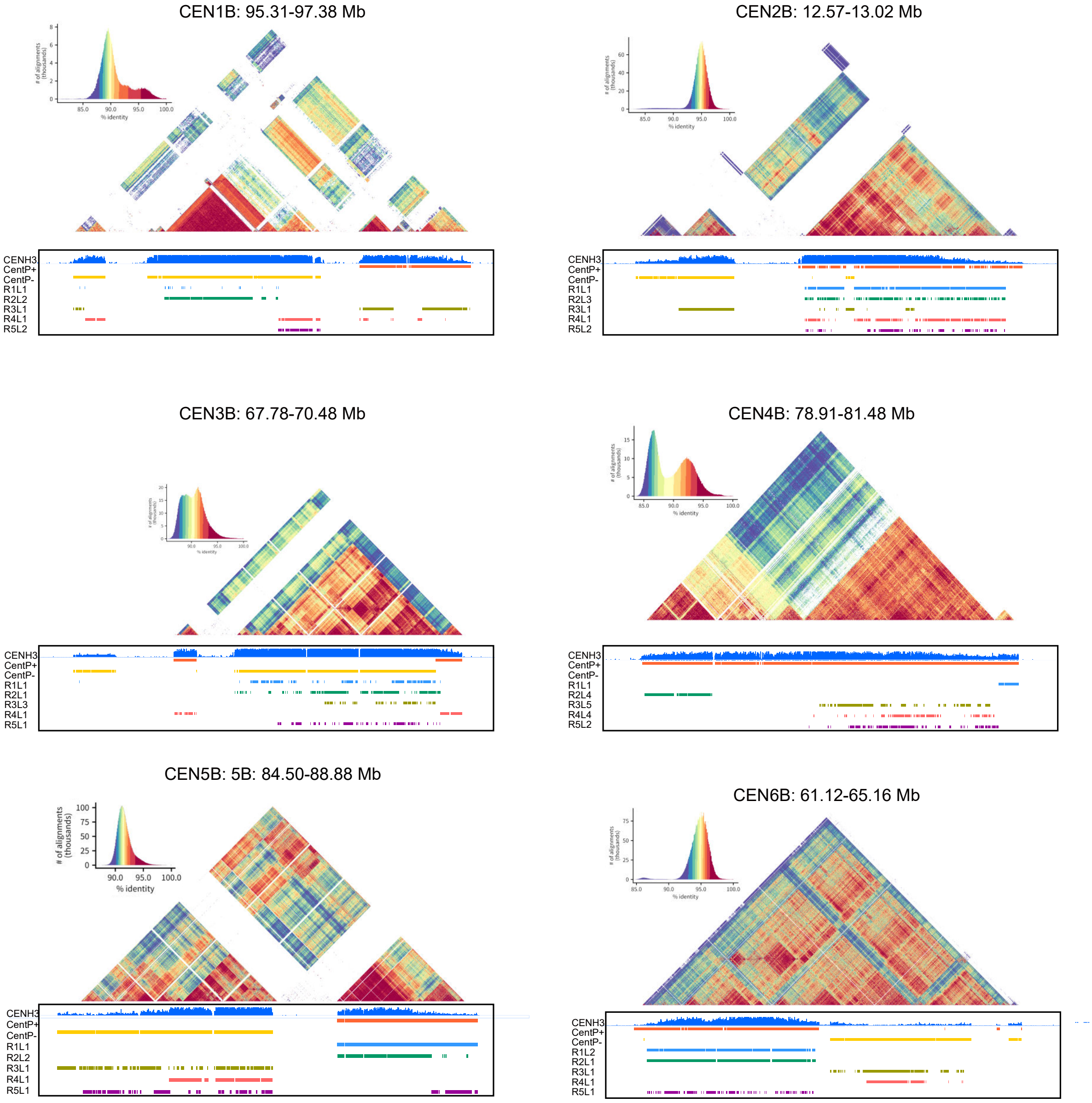

Fig. S13 Higher order structure analysis of CentP satellite DNA arrays in the subgenome B of *P. purpureum* 'Purple'.

StainedGlass sequence identity heatmap of putative centromeric regions of the subgenome B. Histograms at the top left show the assignment of colors to sequence identity values for each heatmap.

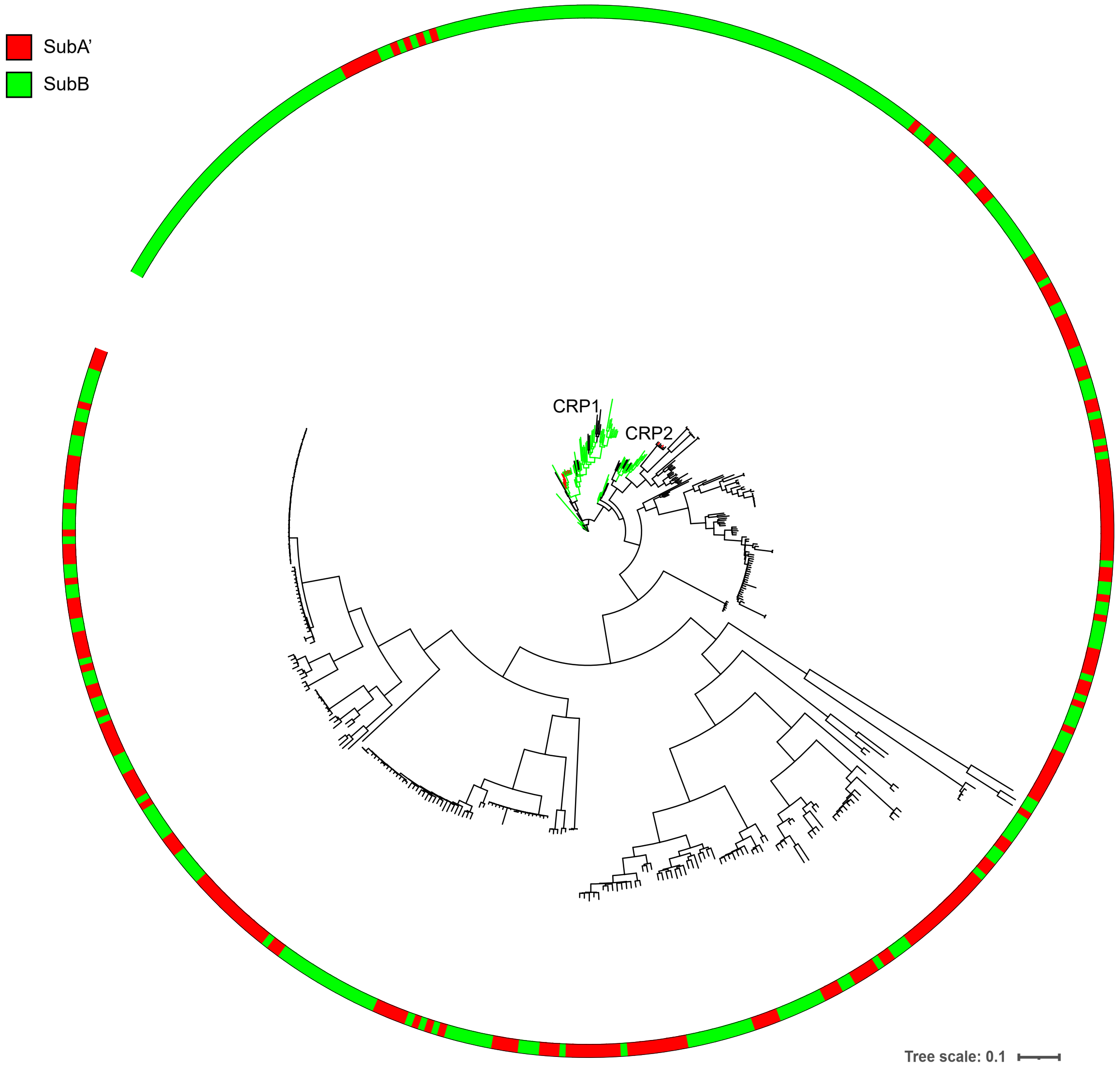

Fig. S14 Phylogenetic tree for Centromeric Retrotransposon in *Pennisetum* (CRPs) in the subgenomes of *P. purpureum* 'Purple'.

Phylogenetic tree for CRPs in the SubA' (red) and SubB (green) of *P. purpureum* 'Purple'.

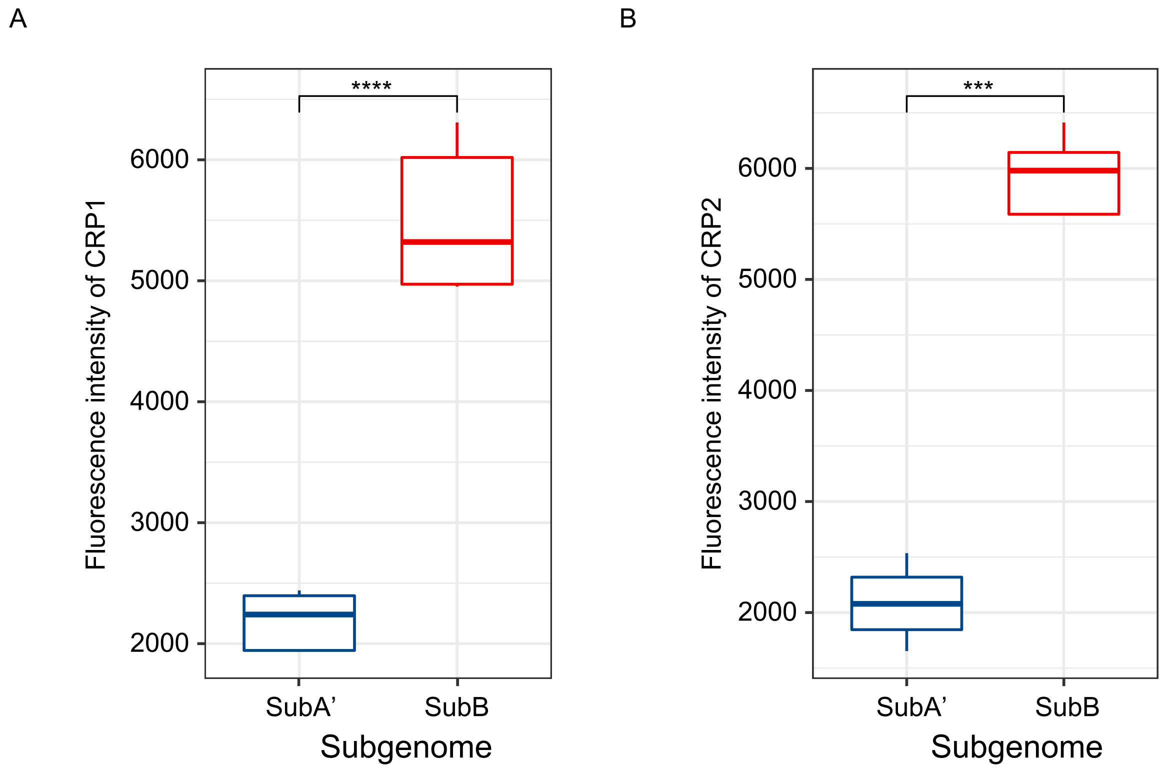

Fig. S15 Fluorescence intensity of CRP1 and CRP2 in the two subgenomes of *P. purpureum* 'Purple'. The y-axis represents the fluorescence intensity of CRP1 and CRP2 in the two subgenomes of *P. purpureum* 'Purple'. The x-axis represents the two subgenomes of *P. purpureum* 'Purple'. The x-axis represents the two subgenomes of *P. purpureum* 'Purple'. ***P < 0.001, ***P < 0.0001, Student's t-test.

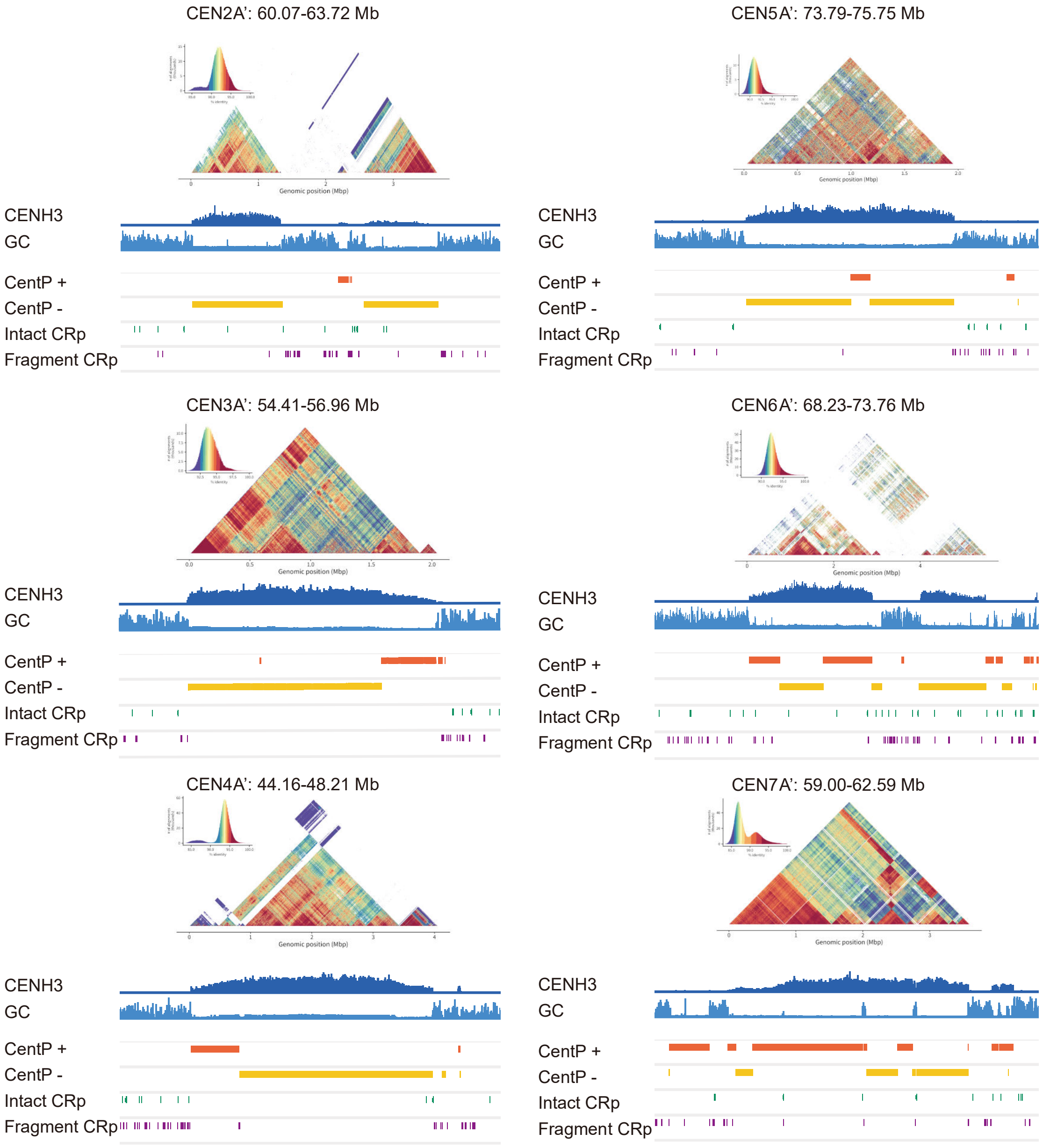

Fig. S16 The distribution of CRPs in the centromeres of SubA'. StamnedGlass sequence identity heatmap of putative centromeric regions of the subgenome A'. Histograms at the top left show the assignment of colors to sequence identity values for each heatmap.

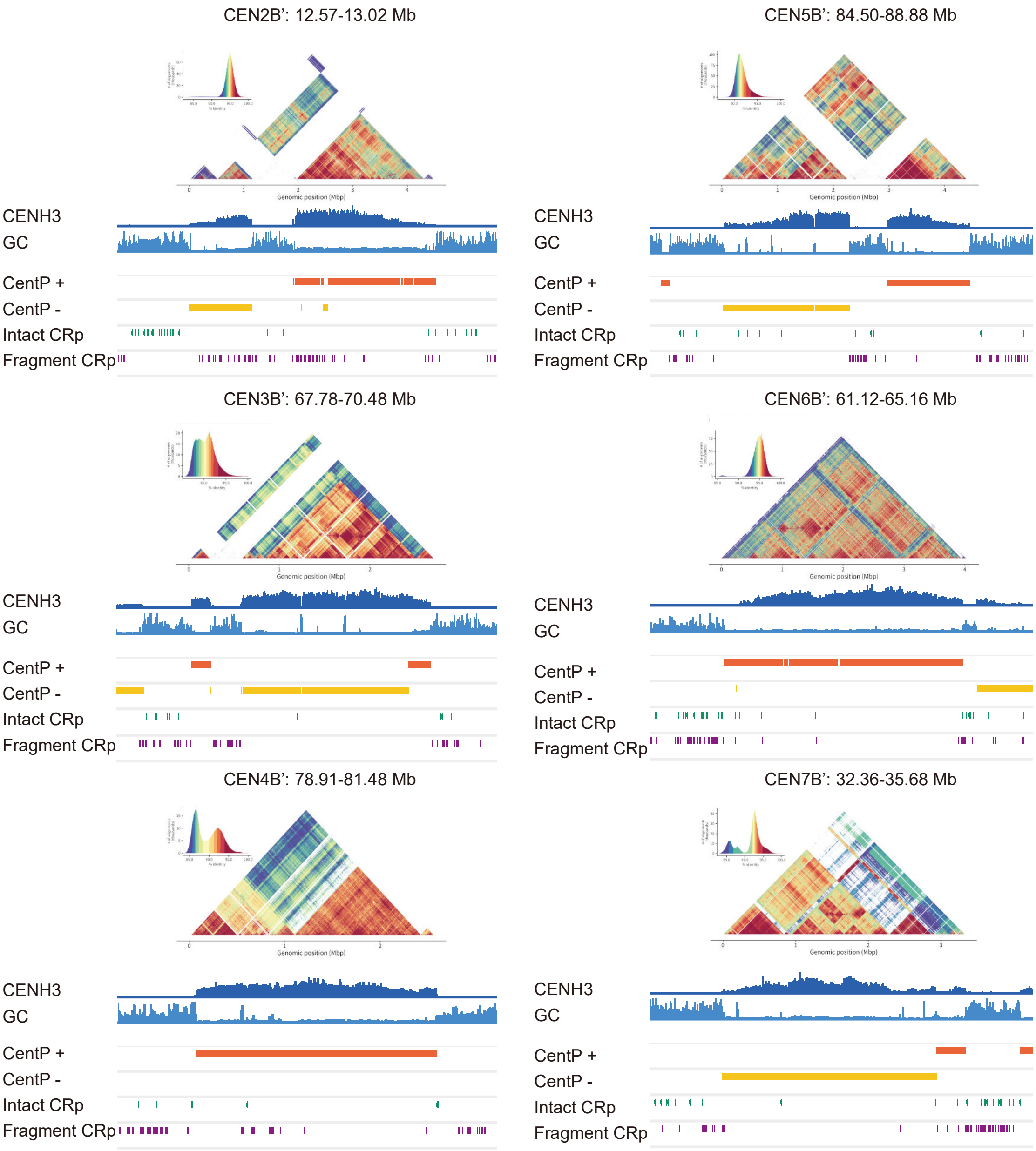

Fig. S17 The distribution of CRPs in the centromeres of SubB. StamnedGlass sequence identity heatmap of putative centromeric regions of the subgenome B. Histograms at the top left show the assignment of colors to sequence identity values for each heatmap.

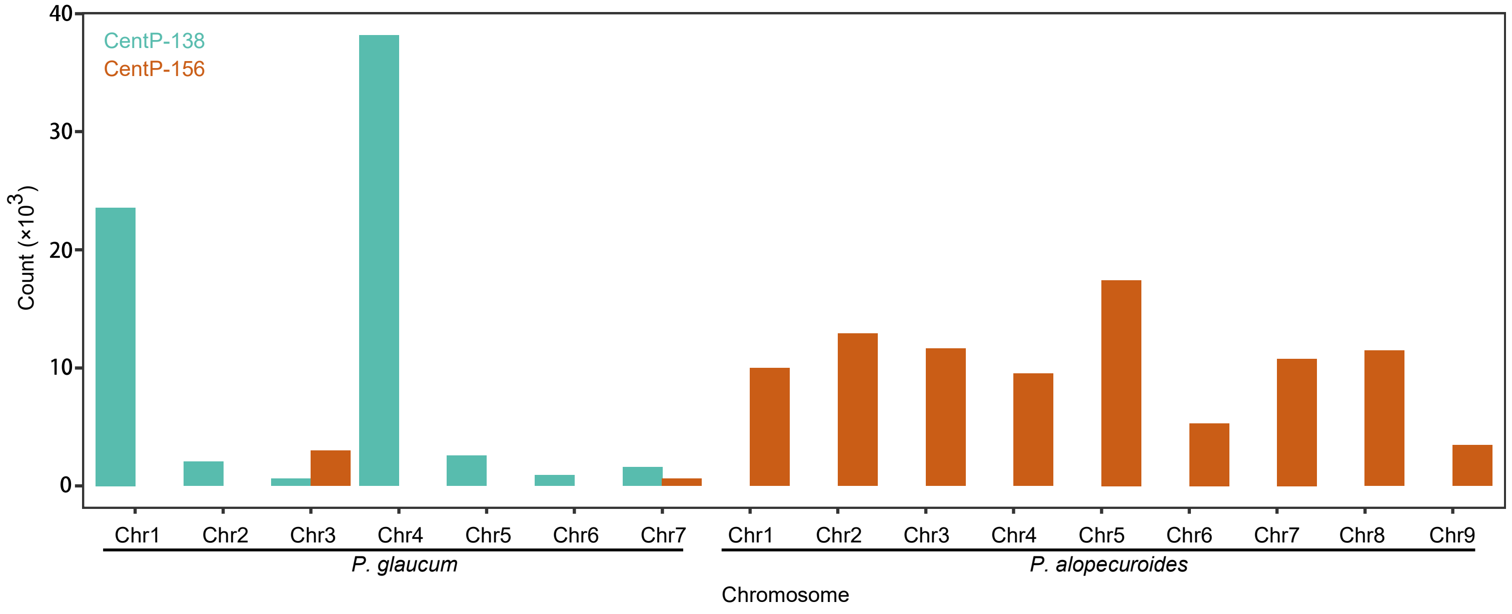

Fig. S18 CentP count in all the chromosomes in *P. glaucum* (2n = 2x = 14, x = 7) and *P. alopecuroides* (2n = 2x = 18, x = 9).

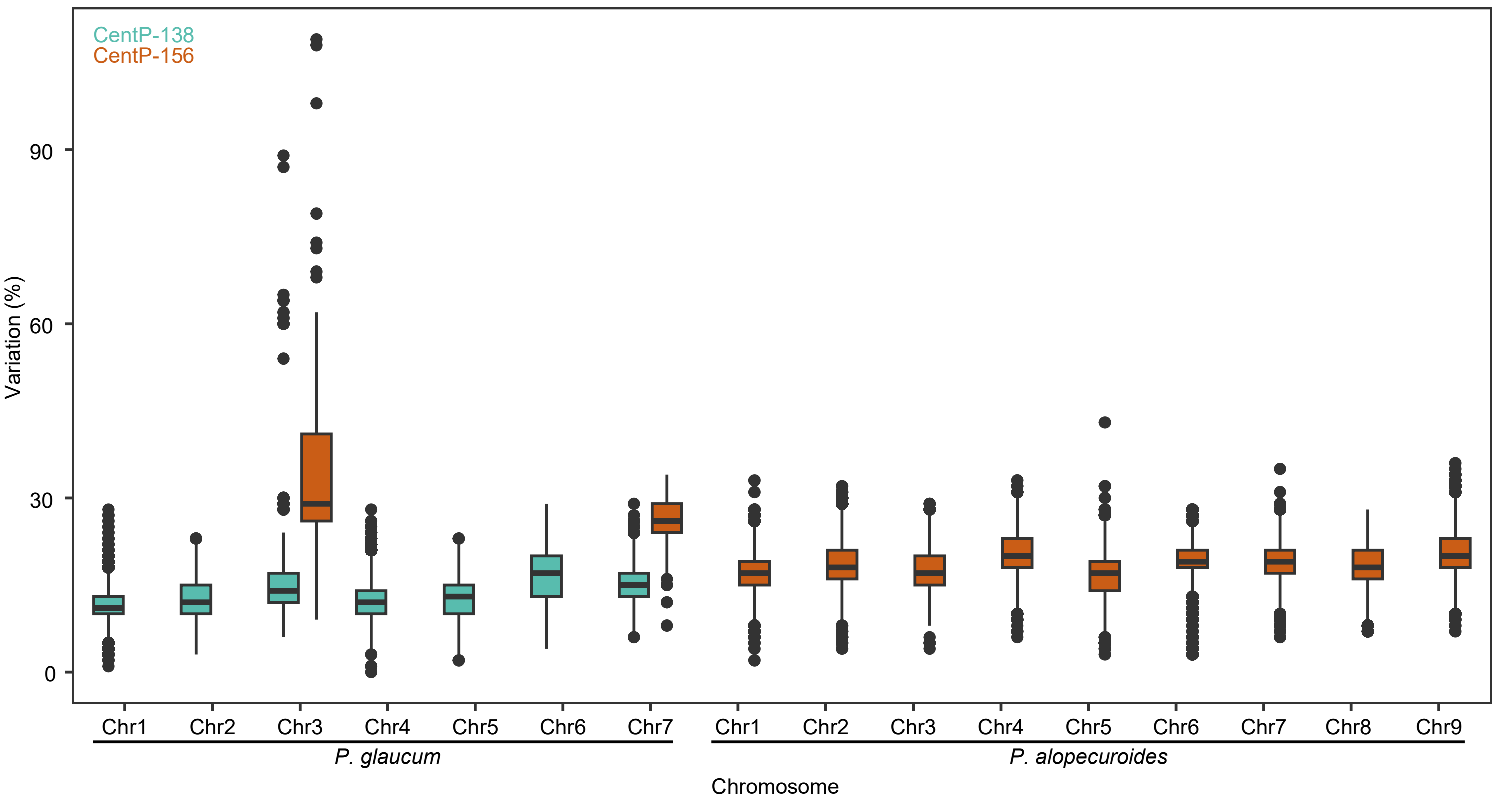

Fig. S19 Sequence variation of CentP monomer in all the chromosomes in *P. glaucum* (2n = 2x = 14, x = 7) and *P. alopecuroides* (2n = 2x = 18, x = 9).

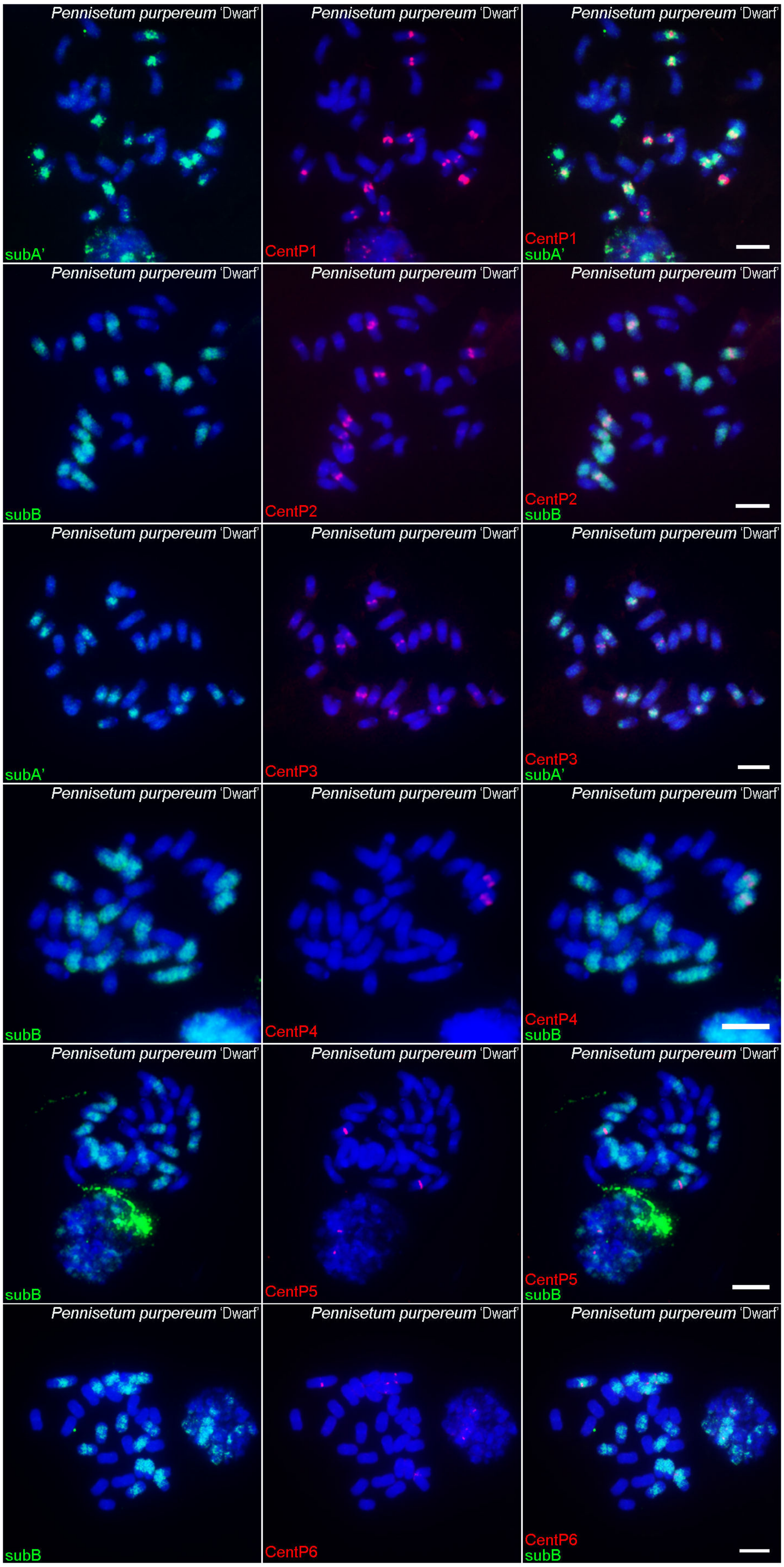

Fig. S20 FISH mapping of six CentP monomers in *P. purpureum* 'Dwarf'. Chromosomes counterstained with DAPI. FISH signals of six CentP probes in red color, and corresponding subgenome-specific abundant LTR-RTs in green color. Scale bar = 5 µm.

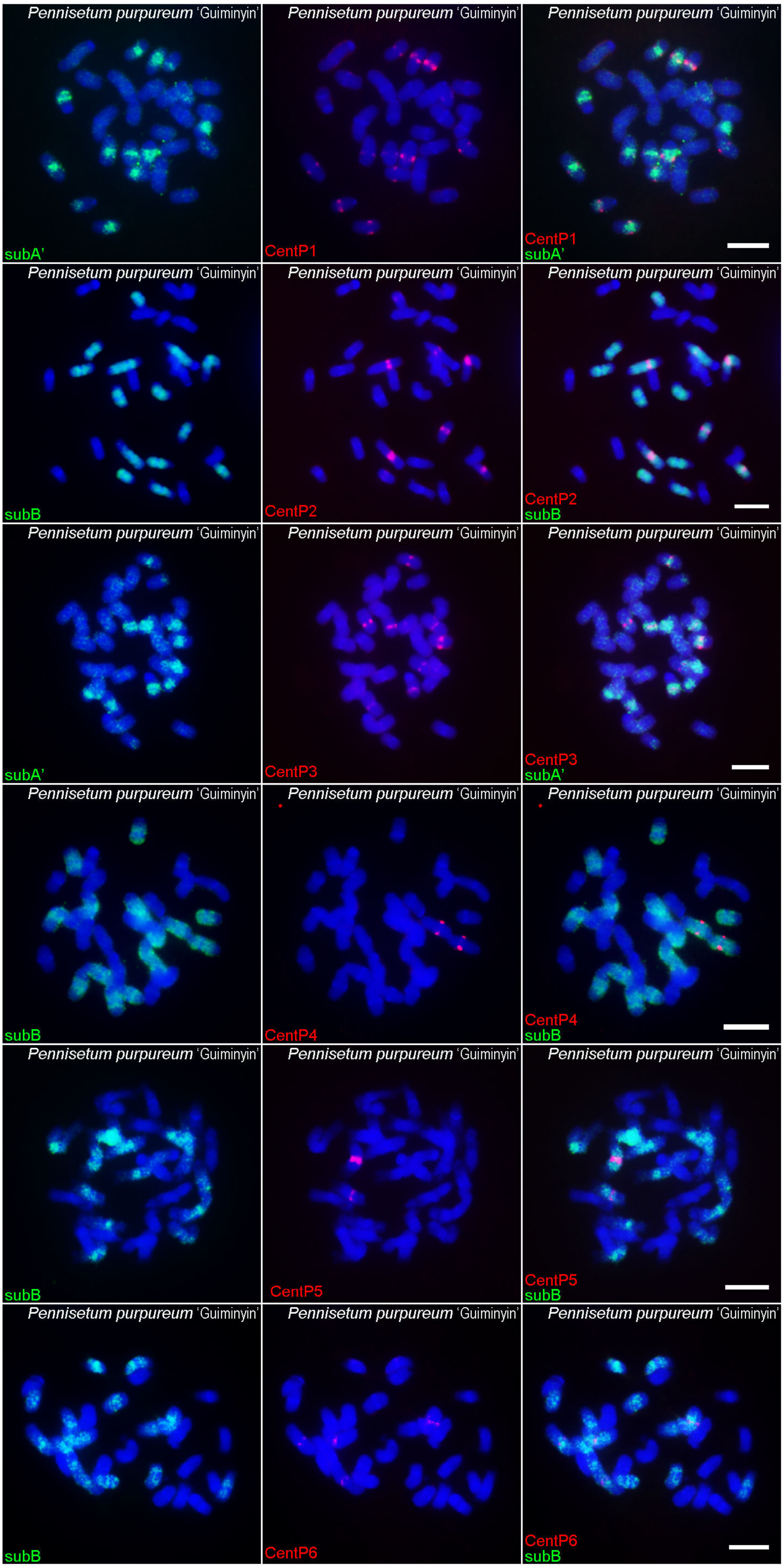

Fig. S21 FISH mapping of six CentP monomers in *P. purpureum* 'Guiminyin'. Chromosomes counterstained with DAPI. FISH signals of six CentP probes in red color, and corresponding subgenome-specific abundant LTR-RTs in green color. Scale bar = 5 μm.

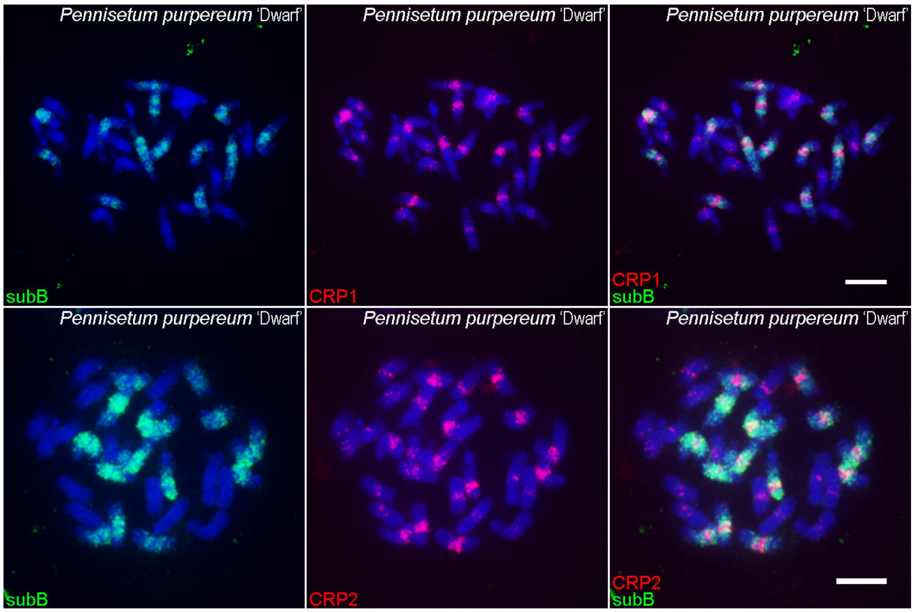

Fig. S22 FISH mapping of two CRPs in *P. purpureum* 'Dwarf'.
Chromosomes counterstained with DAPI. FISH signals oftwo CRP probes in red color, and corresponding subgenome B-specific abundant LTR-RT SubB-Retand1 in green color. Scale bar = 5 µm.

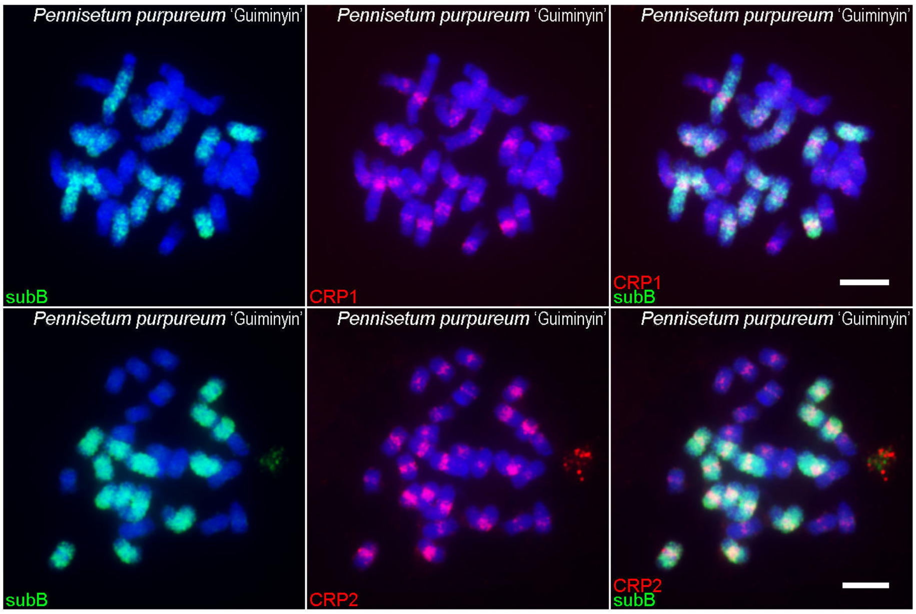

Fig. S23 FISH mapping of two CRPs in *P. purpureum* 'Guiminyin'. Chromosomes counterstained with DAPI. FISH signals oftwo CRP probes in red color, and corresponding subgenome B-specific abundant LTR-RT SubB-Retand1 in green color. Scale bar = 5 μm.

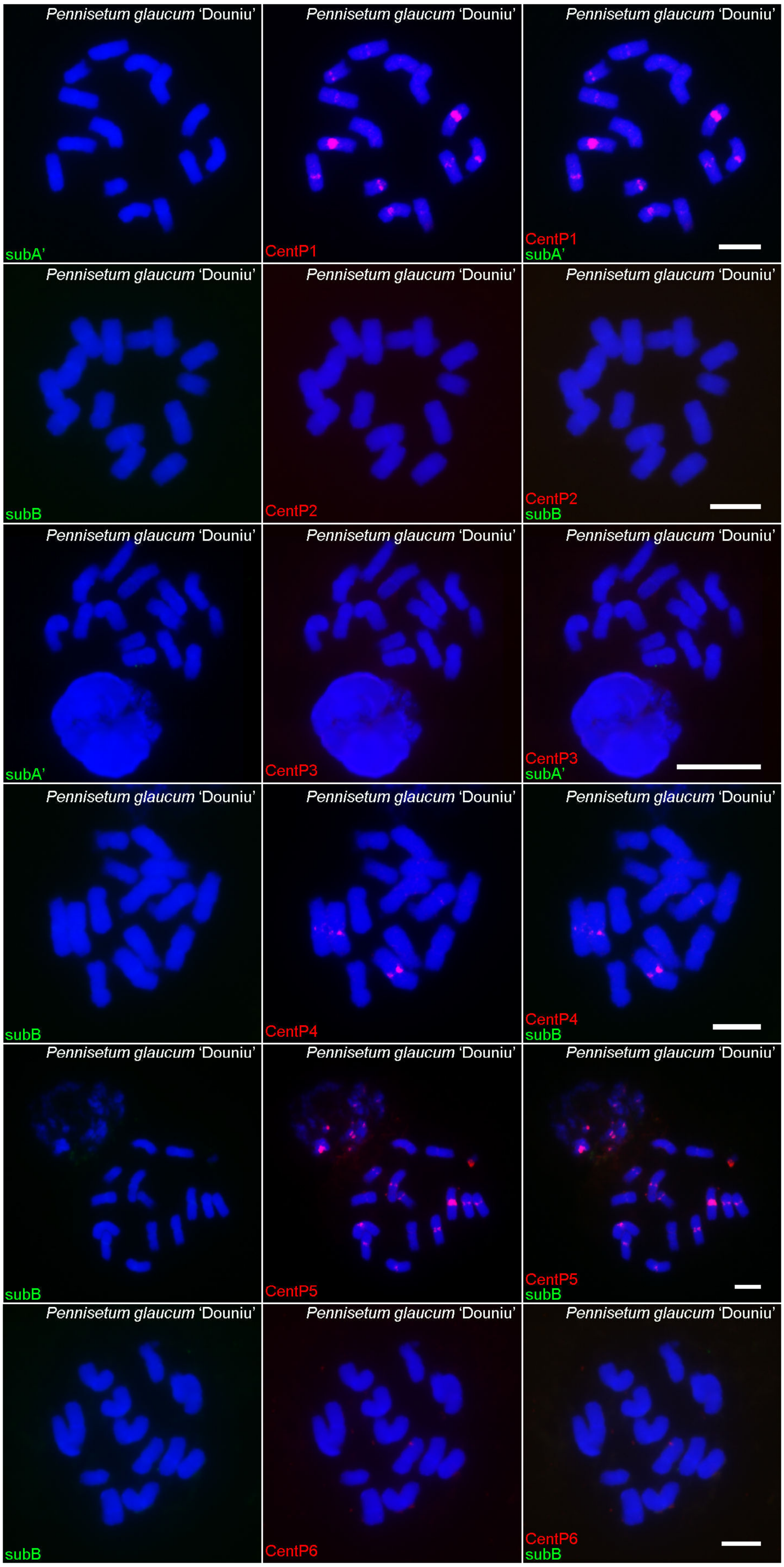

Fig. S24 FISH mapping of six CentP monomers in *P. glaucum* 'Douniu'. Chromosomes counterstained with DAPI. FISH signals of six CentP probes in red color, and corresponding subgenome-specific abundant LTR-RTs in green color. Scale bar = 5 μm.

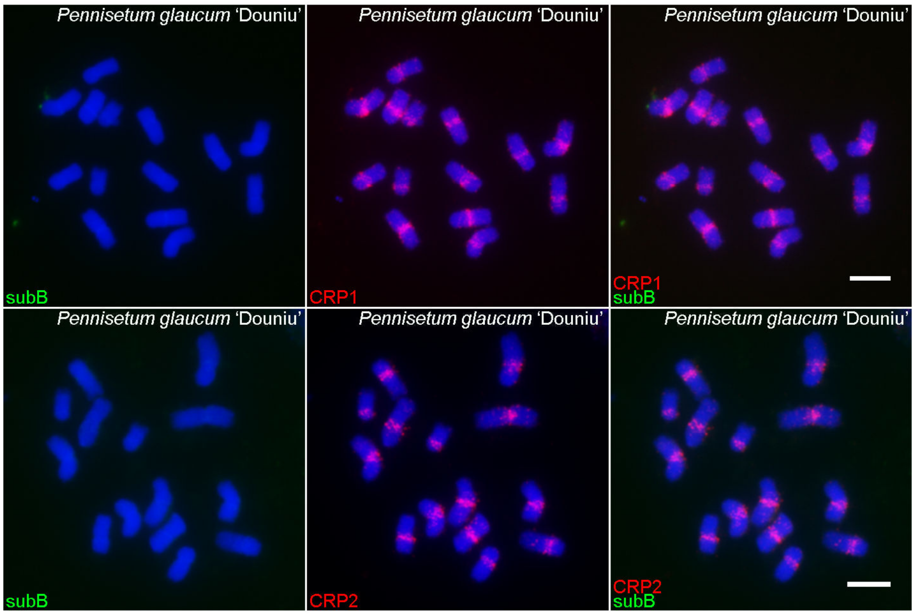

Fig. S25 FISH mapping of two CRPs in *P. glaucum* 'Douniu'.
Chromosomes counterstained with DAPI. FISH signals oftwo CRP probes in red color, and corresponding subgenome B-specific abundant LTR-RT SubB-Retand1 in green color. Scale bar = 5 μm.

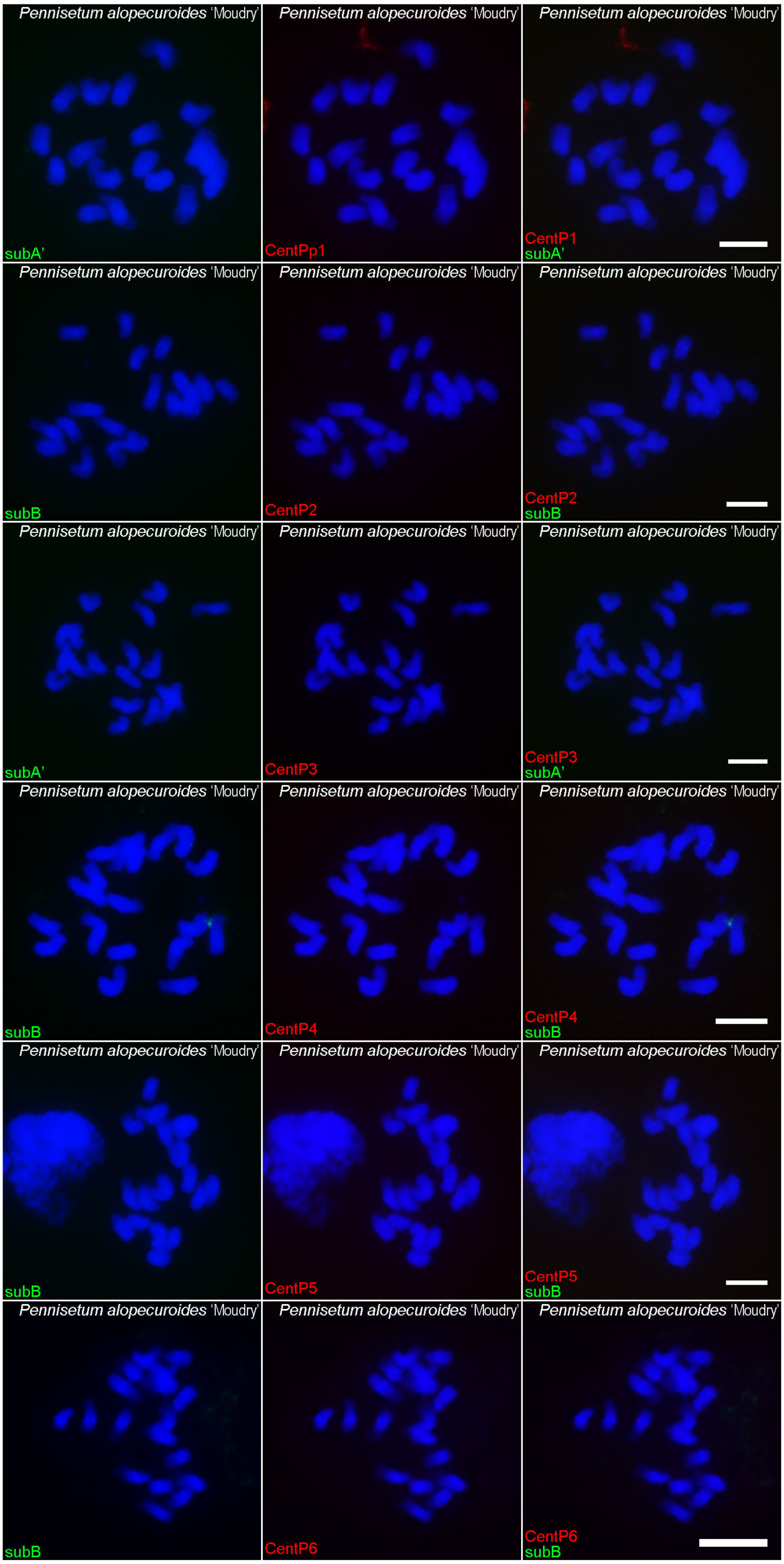

Fig. S26 FISH mapping of six CentP monomers in *P. alopecuroides* 'Moudry'. Chromosomes counterstained with DAPI. FISH signals of six CentP probes in red color, and corresponding subgenome-specific abundant LTR-RTs in green color. Scale bar = 5 μm.

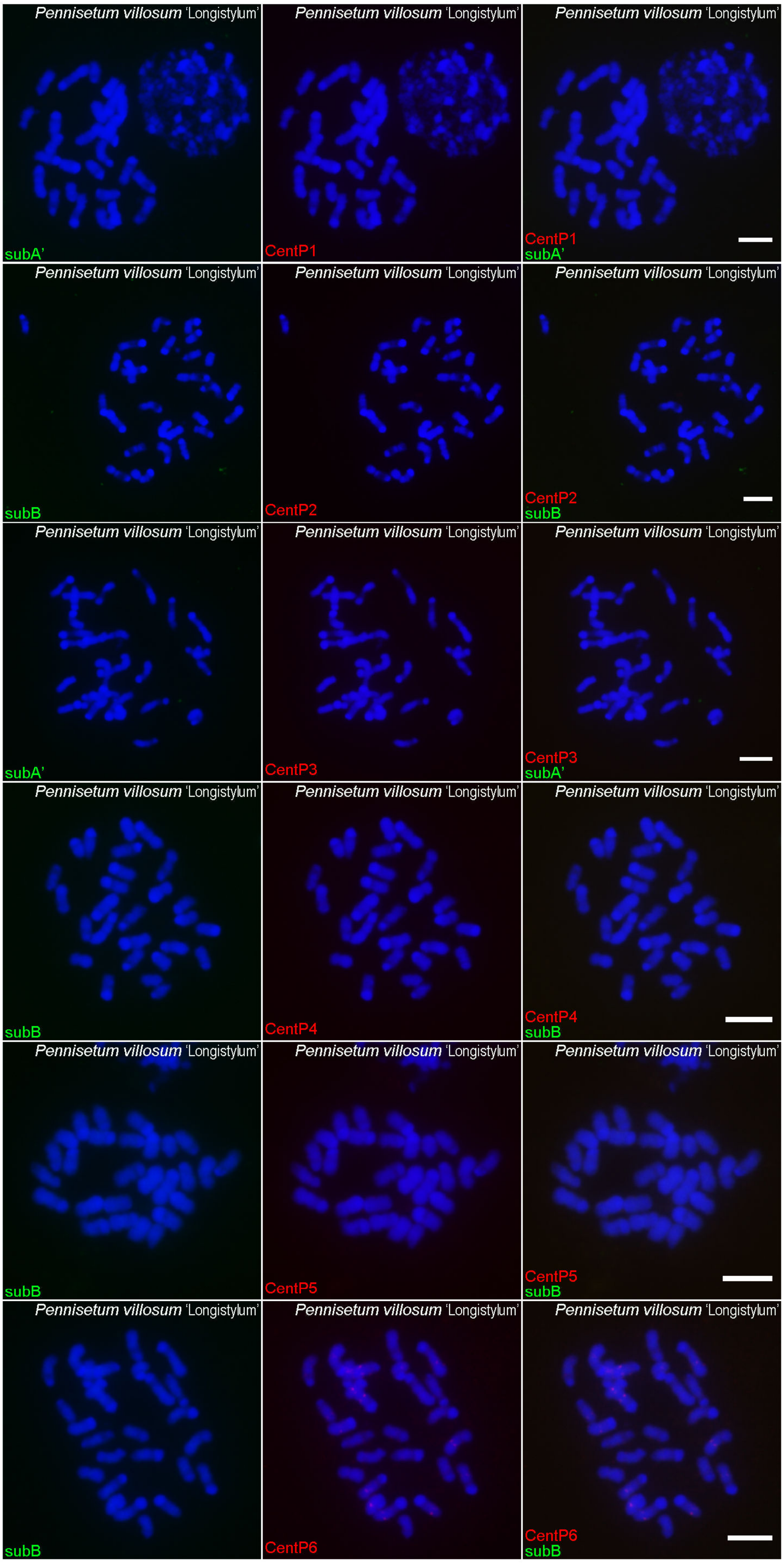

Fig. S27 FISH mapping of six CentP monomers in *P. villosum* 'Longistylum'. Chromosomes counterstained with DAPI. FISH signals of six CentP probes in red color, and corresponding subgenome-specific abundant LTR-RTs in green color. Scale bar = 5 μm.

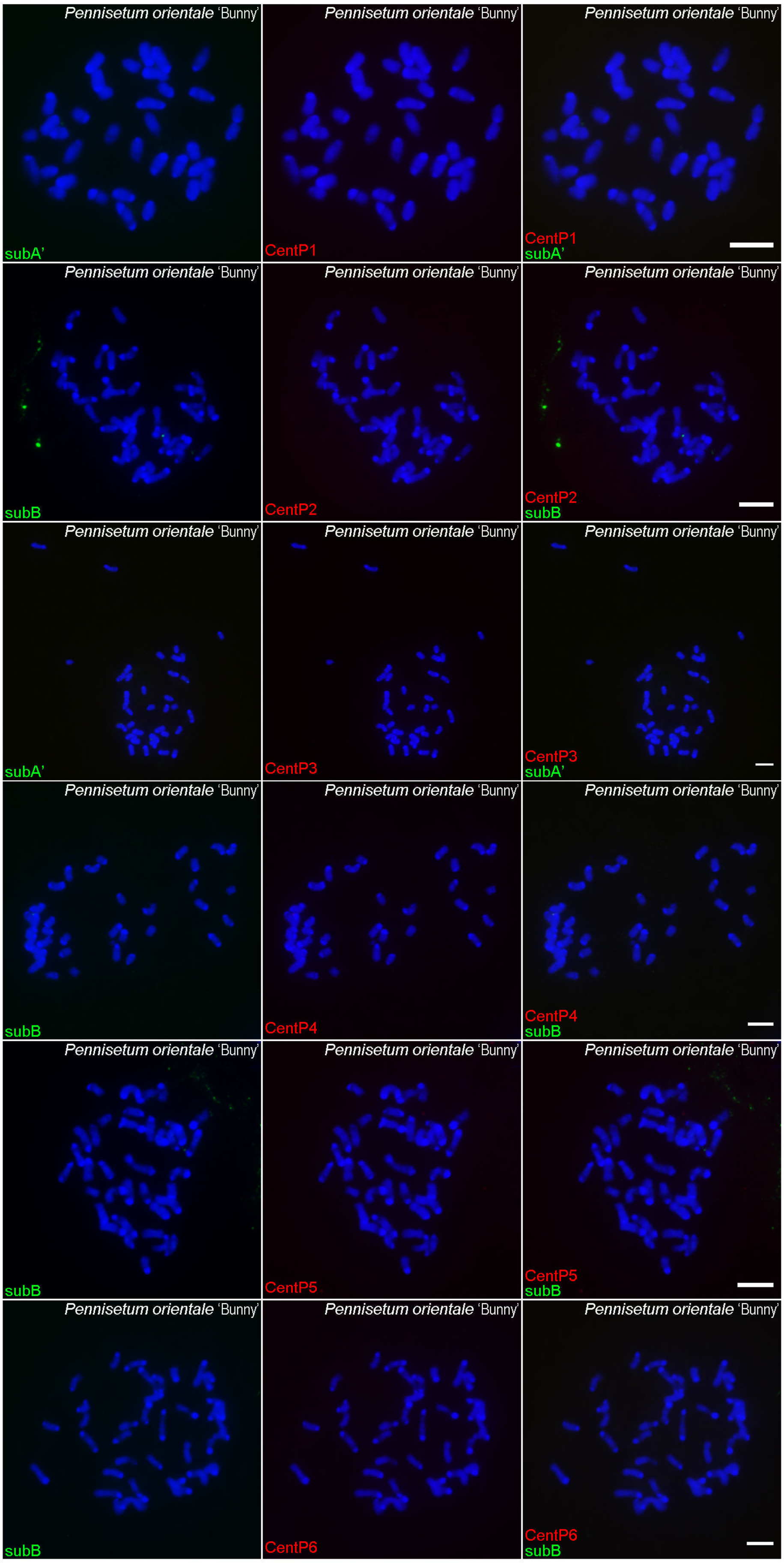

Fig. S28 FISH mapping of six CentP monomers in *P. orientale* 'Bunny'. Chromosomes counterstained with DAPI. FISH signals of six CentP probes in red color, and corresponding subgenome-specific abundant LTR-RTs in green color. Scale bar = 5 µm.

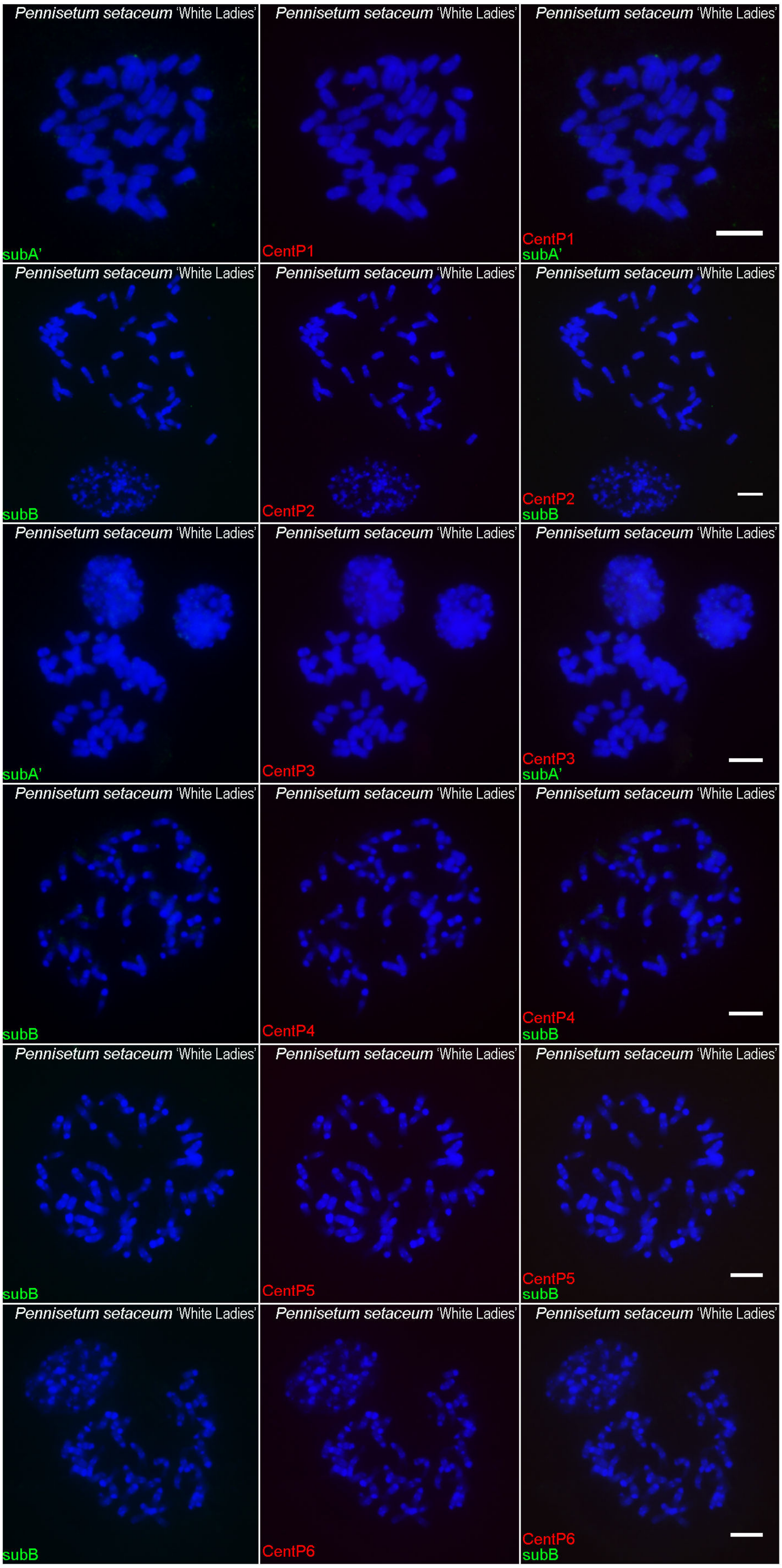

Fig. S29 FISH mapping of six CentP monomers in *P. setaceum* 'White Ladies'. Chromosomes counterstained with DAPI. FISH signals of six CentP probes in red color, and corresponding subgenome-specific abundant LTR-RTs in green color. Scale bar = 5 μm.

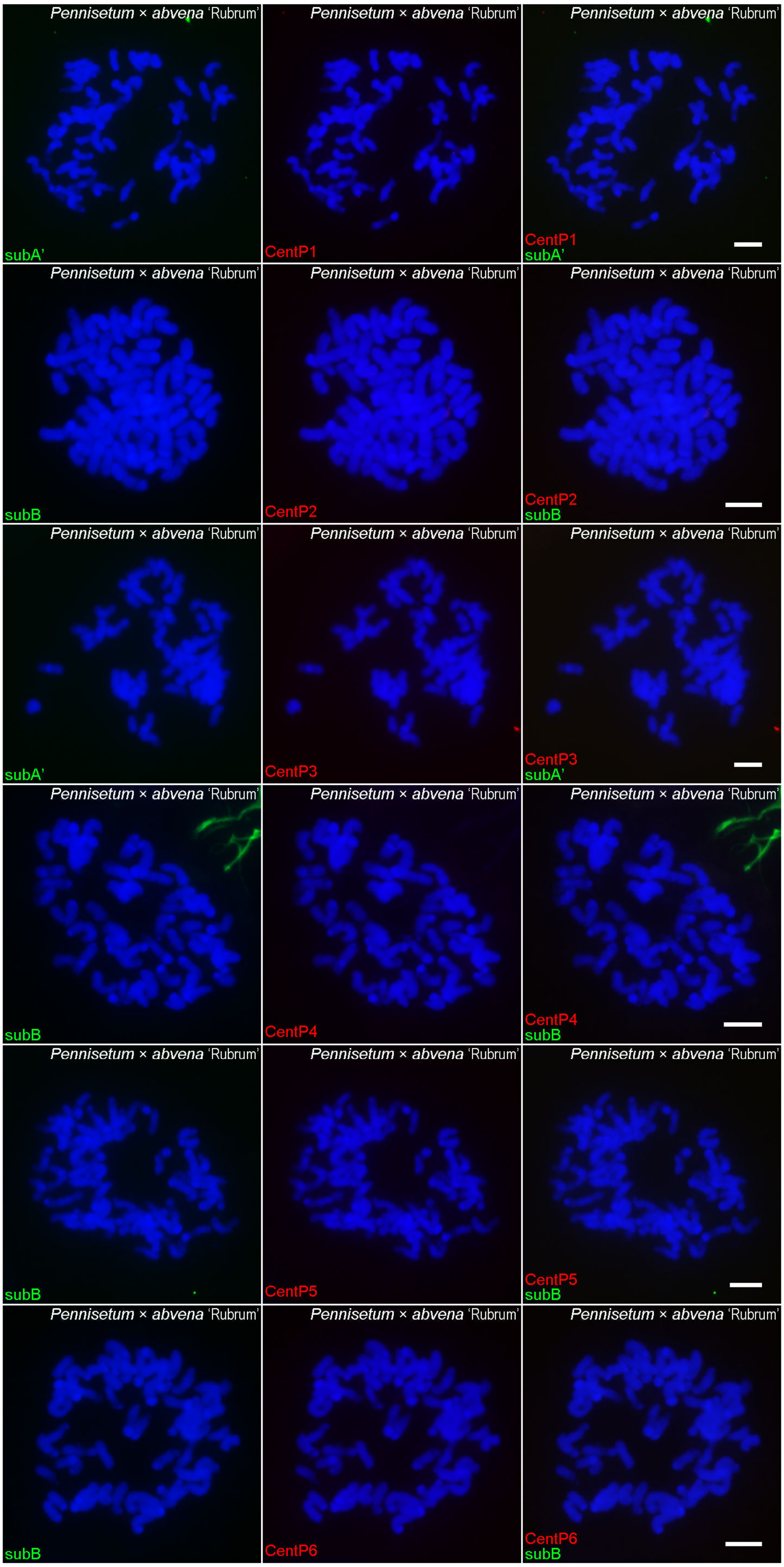

Fig. S30 FISH mapping of six CentP monomers in *P.* × *abvena* 'Rubrum'. Chromosomes counterstained with DAPI. FISH signals of six CentP probes in red color, and corresponding subgenome-specific abundant LTR-RTs in green color. Scale bar = 5 μm.

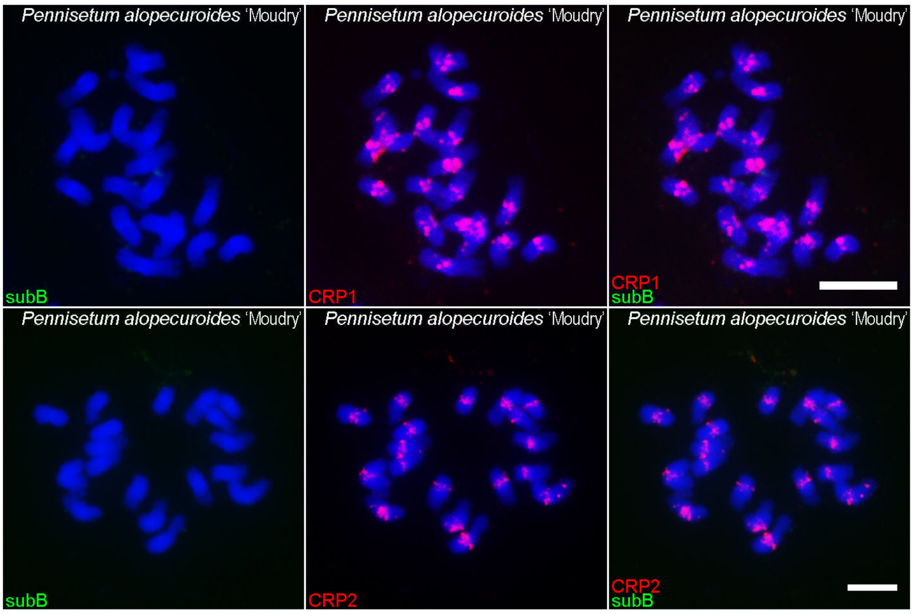

Fig. S31 FISH mapping of two CRPs in *P. alopecuroides* 'Moudry'. Chromosomes counterstained with DAPI. FISH signals of two CRP probes in red color, and corresponding subgenome B-specific abundant LTR-RT SubB-Retand1 in green color. Scale bar = 5 µm.

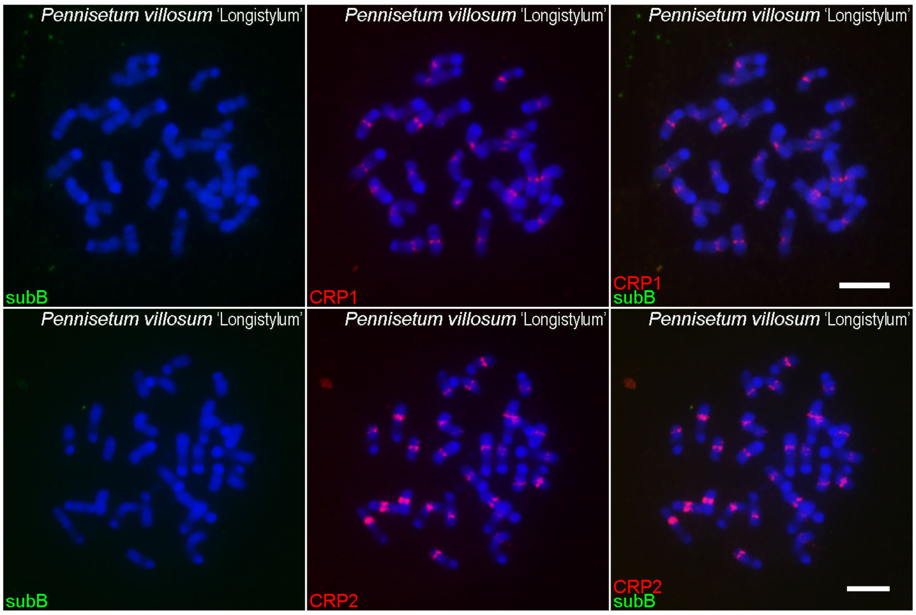

Fig. S32 FISH mapping of two CRPs in *P. villosum* 'Longistylum'. Chromosomes counterstained with DAPI. FISH signals oftwo CRP probes in red color, and corresponding subgenome B-specific abundant LTR-RT SubB-Retand1 in green color. Scale bar = 5 μm.

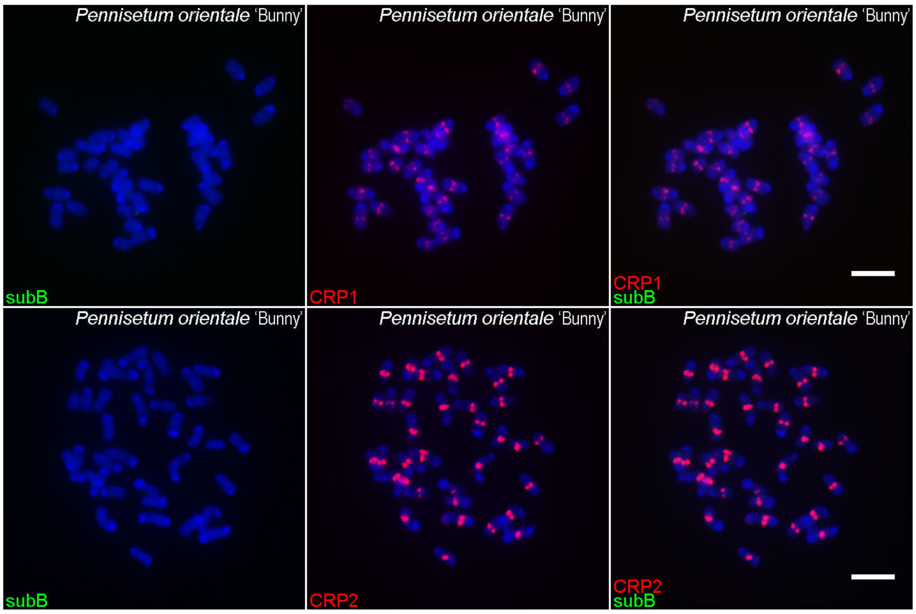

Fig. S33 FISH mapping of two CRPs in *P. orientale* 'Bunny'.
Chromosomes counterstained with DAPI. FISH signals oftwo CRP probes in red color, and corresponding subgenome B-specific abundant LTR -RT SubB-Retand1 in green color. Scale bar = 5 μm.

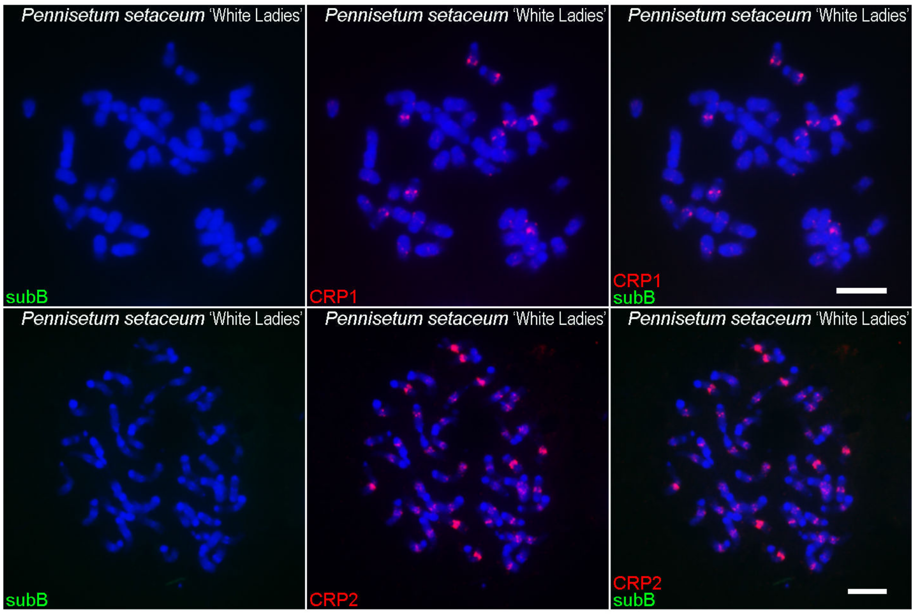

Fig. S34 FISH mapping of two CRPs in *P. setaceum* 'White Ladies'. Chromosomes counterstained with DAPI. FISH signals oftwo CRP probes in red color, and corresponding subgenome B-specific abundant LTR-RT SubB-Retand1 in green color. Scale bar = 5 μm.

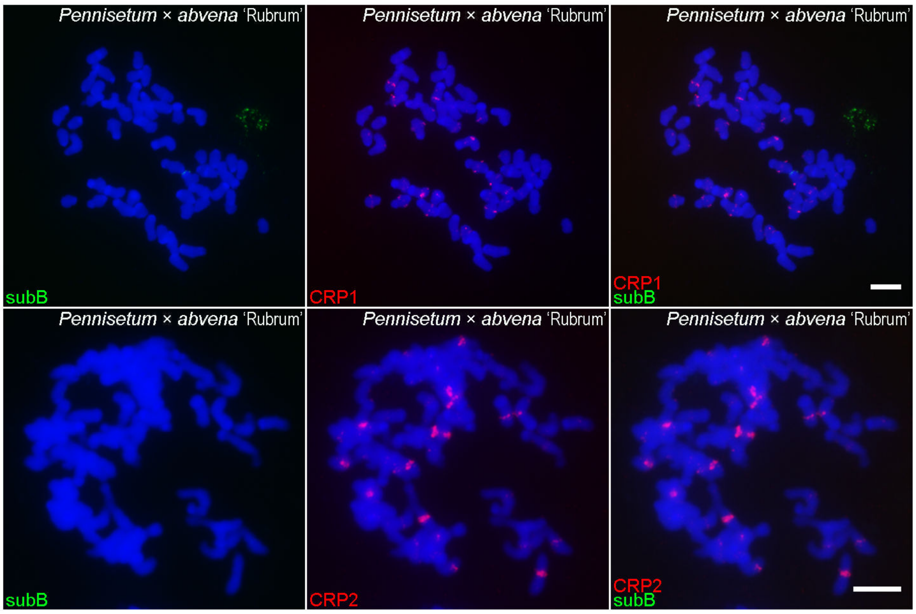

Fig. S35 FISH mapping of two CRPs in *P.* × *abvena* 'Rubrum'.
Chromosomes counterstained with DAPI. FISH signals oftwo CRP probes in red color, and corresponding subgenome B-specific abundant LTR-RT SubB-Retand1 in green color. Scale bar = 5 µm.
